# Phylogeography and Antigenic Diversity of Low-Pathogenic Avian Influenza H13 and H16 Viruses

**DOI:** 10.1128/JVI.00537-20

**Published:** 2020-06-16

**Authors:** Josanne H. Verhagen, Marjolein Poen, David E. Stallknecht, Stefan van der Vliet, Pascal Lexmond, Srinand Sreevatsan, Rebecca L. Poulson, Ron A. M. Fouchier, Camille Lebarbenchon

**Affiliations:** a Erasmus Medical Center, Department of Viroscience, Rotterdam, The Netherlands; b Linnaeus University, Department of Biology and Environmental Science, Kalmar, Sweden; c Southeastern Cooperative Wildlife Disease Study, College of Veterinary Medicine, Department of Population Health, University of Georgia, Athens, Georgia, USA; d Michigan State University, College of Veterinary Medicine, Department of Pathobiology and Diagnostic Investigation, East Lansing, Michigan, USA; e Université de La Réunion, UMR Processus Infectieux en Milieu Insulaire Tropical, INSERM 1187, CNRS 9192, IRD 249, Sainte-Clotilde, La Réunion, France; Cornell University

**Keywords:** avian viruses, influenza, evolution, epidemiology, ecology, antigenic variation, seabird, gulls, wild birds

## Abstract

Low-pathogenic avian influenza viruses (LPAIVs) are genetically highly variable and have diversified into multiple evolutionary lineages that are primarily associated with wild-bird reservoirs. Antigenic variation has been described for mammalian influenza viruses and for highly pathogenic avian influenza viruses that circulate in poultry, but much less is known about antigenic variation of LPAIVs. In this study, we focused on H13 and H16 LPAIVs that circulate globally in gulls. We investigated the evolutionary history and intercontinental gene flow based on the hemagglutinin (HA) gene and used representative viruses from genetically distinct lineages to determine their antigenic properties by hemagglutination inhibition assays. For H13, at least three distinct genetic clades were evident, while for H16, at least two distinct genetic clades were evident. Twenty and ten events of intercontinental gene flow were identified for H13 and H16 viruses, respectively. At least two antigenic variants of H13 and at least one antigenic variant of H16 were identified. Amino acid positions in the HA protein that may be involved in the antigenic variation were inferred, and some of the positions were located near the receptor binding site of the HA protein, as they are in the HA protein of mammalian influenza A viruses. These findings suggest independent circulation of H13 and H16 subtypes in gull populations, as antigenic patterns do not overlap, and they contribute to the understanding of the genetic and antigenic variation of LPAIVs naturally circulating in wild birds.

**IMPORTANCE** Wild birds play a major role in the epidemiology of low-pathogenic avian influenza viruses (LPAIVs), which are occasionally transmitted—directly or indirectly—from them to other species, including domestic animals, wild mammals, and humans, where they can cause subclinical to fatal disease. Despite a multitude of genetic studies, the antigenic variation of LPAIVs in wild birds is poorly understood. Here, we investigated the evolutionary history, intercontinental gene flow, and antigenic variation among H13 and H16 LPAIVs. The circulation of subtypes H13 and H16 seems to be maintained by a narrower host range, in particular gulls, than the majority of LPAIV subtypes and may therefore serve as a model for evolution and epidemiology of H1 to H12 LPAIVs in wild birds. The findings suggest that H13 and H16 LPAIVs circulate independently of each other and emphasize the need to investigate within-clade antigenic variation of LPAIVs in wild birds.

## Abstract (precis)

Wild birds play a major role in the epidemiology of low-pathogenic avian influenza viruses (LPAIVs), which are occasionally transmitted—directly or indirectly—from them to other species, including domestic animals, wild mammals, and humans, where they can cause subclinical to fatal disease. Despite a multitude of genetic studies, the antigenic variation of LPAIVs in wild birds is poorly understood. Here, we investigated the evolutionary history, intercontinental gene flow, and antigenic variation among H13 and H16 LPAIVs. The circulation of subtypes H13 and H16 seems to be maintained by a narrower host range, in particular gulls, than the majority of LPAIV subtypes and may therefore serve as a model for evolution and epidemiology of H1 to H12 LPAIVs in wild birds. The findings suggest that H13 and H16 LPAIVs circulate independently of each other and emphasize the need to investigate within-clade antigenic variation of LPAIVs in wild birds.

## INTRODUCTION

Wild birds of the orders Anseriformes (mainly ducks, geese, and swans) and Charadriiformes (mainly gulls, terns, and waders) play a major role in the epidemiology of low-pathogenic avian influenza viruses (LPAIVs). LPAIVs are characterized into subtypes based on the surface proteins hemagglutinin (HA; H1 to H16) and neuraminidase (NA; N1 to N9), e.g., H13N6. Ducks play an important role in the epidemiology of most LPAIV subtypes. However, birds of the order Charadriiforme*s*—in particular gulls— are the major reservoir for subtypes H13 and H16 (see Table S1 in the supplemental material) (1–4). High prevalence of H13 and/or H16 LPAIVs has been observed in juvenile gulls at breeding colony sites (5–7) and in adults during spring and/or fall migration (8, 9). H13 and H16 viruses have a global distribution. Since their first detection in 1977, H13 viruses have been detected in North America, South America, Europe, Asia, Africa, and Oceania. Since their first detection in 1975, H16 viruses have been detected in North America, South America, Europe, and Asia. The spatial isolation of host populations has shaped LPAIV evolution and led to the independent circulation of different virus gene pools between the Western and Eastern hemispheres (10). However, some pelagic gull populations connect multiple continents through seasonal migration and overlapping distributions and could facilitate rapid and long-distance dispersal of LPAIV genomes (2, 9, 11–14). For instance, great black-backed gulls (Larus marinus) migrate between Europe and the east coast of North America, and LPAIVs consisting of both North American and Eurasian genes have been isolated from this species (12). Upon intercontinental gene flow, i.e., the movement of genes between the different continents, some LPAIV genes seem to have become established in the population, e.g., H6 (15).

Influenza A viruses (IAVs) belong to the family *Orthomyxoviridae* and are negative-sense single-stranded RNA viruses with a segmented genome. The genome consists of eight segments encoding 12 proteins or more, including the surface proteins HA and NA. The HA protein of IAV is a major determinant for virus binding to cells and subsequent cell entry and for generation of IAV-specific antibodies, and it is thus subjected to strong selective pressure (16). Indeed, in wild birds—in particular mallards (Anas platyrhynchos)—LPAIV infection dynamics between LPAIV subtypes seem to be shaped partially by preexisting homo- or heterologous antibodies (17). Furthermore, within other host systems, evasion of IAV-specific antibodies by IAVs—called antigenic variation—has been described for seasonal human IAVs (18, 19), swine IAVs (20–22), and equine IAVs (23) and for highly pathogenic avian influenza viruses (HPAIVs) that circulate in poultry (24, 25). Despite numerous studies on the genetic variation of LPAIVs in wild birds, the antigenic variation within LPAIV subtypes that circulate in wild birds has barely been investigated (26, 27).

To better understand LPAIV epidemiology in gulls, we investigated the global distribution of H13 and H16 LPAIVs and the antigenic variation of a representative subset of H13 and H16 LPAIVs. Based on the sequencing of HA genes of 84 viruses and hemagglutination inhibition assays, we showed that intercontinental H13 and H16 gene flow occurred frequently and that H16 genetic lineages did not form antigenic clusters, suggesting that clade-defining mutations were not in critical epitopes (i.e., part of the antigen that binds to specific antibodies). In contrast, the H13 genetic clades partially corresponded with the antigenic variation of H13 LPAIVs, suggesting that some of the clade-defining mutations were in critical epitopes.

## RESULTS

### Phylogeographic structure and intercontinental gene flow.

**FIG 1:**
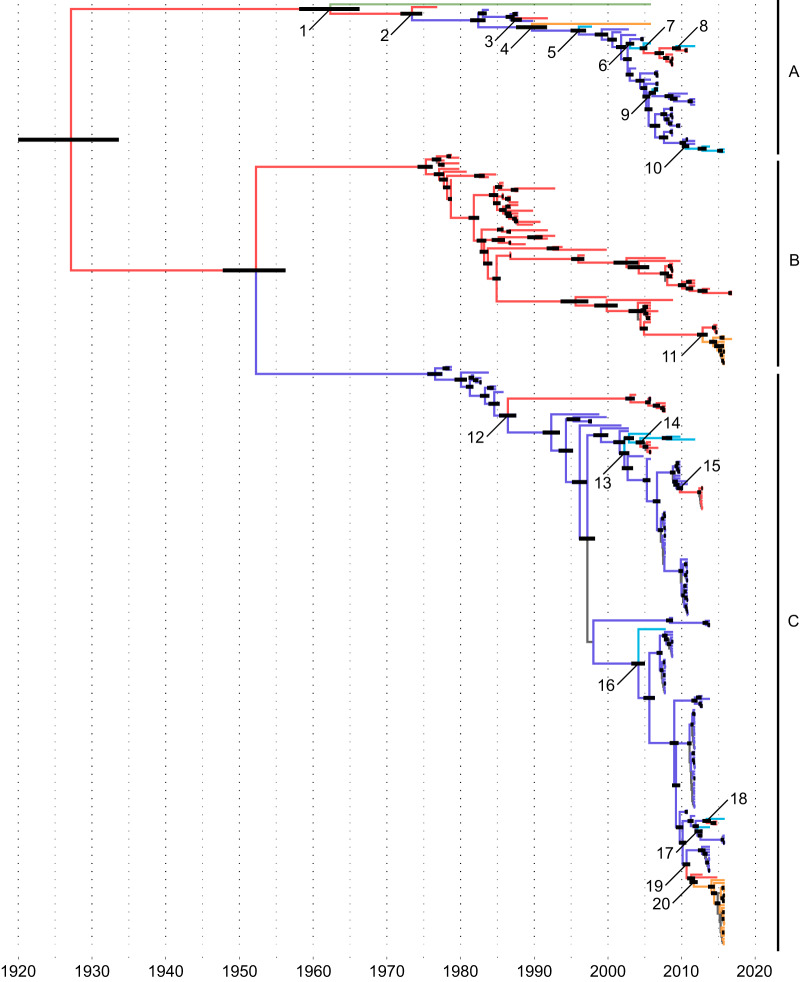
Maximum-clade-credibility (MCC) tree for influenza A virus H13 hemagglutinin subtype (*n* = 338). Branches were colored according to most probable geographic origin (red, North America; orange, South America; dark blue, Europe; light blue, Asia; green, Oceania; gray, not identified). Black node bars represent the 95% highest posterior densities for times to the common ancestors. Numbers highlight intercontinental gene flow events as detailed in Table 1 and Fig. S3. Virus strain names and posterior probabilities are detailed in Fig. S2.

Phylogenetic analyses supported the idea that the H13 HA was structured in three major genetic lineages (lineages A to C) (Fig. 1; also, see Fig. S1 and S2). The time to the most recent common ancestor (tMRCA) of the H13 HA gene was dated to 1927 (±95% highest posterior density [HPD], 1920–1934). The tMRCA of viruses of clade A (1963 [1958–1966]) was longer than those of clade B (1975 [1974–1976]) and C (1977 [1976–1978]). Our analyses support the idea that the geographic origins of H13 viruses of clades B and C could be North America and Europe, respectively (posterior probabilities for the geographic origin of the MRCA, 1 for clade B and 1 for clade C). For clade A, limited historical data on viruses from different locations as well as low posterior probability (0.62) precludes a conclusion on the geographic origin of the MRCA.

Since the first isolation of an H13 IAV from a gull in 1977, 20 potential events of intercontinental gene flow were identified (indicated by the numerals 1 to 20 in Fig. 1 and Table 1 and also in Fig. S3). Clade A supports the maintenance of H13 in European gulls, with evidence of multiple introductions to North America and Asia (events 3, 5, 6, 9, and 10), and a reverse introduction from North America to Asia (event 8). Clade C was also composed mainly of viruses circulating in Europe, with evidence of multiple introductions to North America (events 12, 15, and 19) and Asia (events 13, 16, and 17). The introduction of clade C H13 HA in North America (event 19) was followed by an introduction to South America (event 20). Evidence for intercontinental gene flow among North American H13 IAVs occurred among eastern and western North American isolates (events 3, 12, 15, and 19). Clade B was composed almost exclusively of viruses circulating in North America, although one gene flow event to South America occurred recently (event 11).

**TABLE 1:** Intercontinental gene flow events for influenza A virus H13 hemagglutinin^*a*^

H13 clade	Event	Time of MRCA ± 95% HPD	Geographic origin of the MRCA (posterior density)	Location of introduction
A	1	1963 (1958–1966)	North America (0.62)	Oceania
	2	1974 (1972–1975)	North America (0.73)	Europe
	3	1988 (1987–1989)	Europe (1)	North America
	4	1990 (1988–1991)	Europe (0.82)	South America
	5	1996 (1995–1997)	Europe (0.75)	Asia
	6	2003 (2003–2004)	Europe (1)	Asia
	7	2005 (2004–2005)	Asia (0.48)	North America
	8	2009 (2009–2010)	North America (0.9)	Asia
	9	2006 (2006–2007)	Europe (0.96)	Asia
	10	2011 (2010–2011)	Europe (1)	Asia
B	11	2013 (2012–2014)	North America (0.96)	South America
C	12	1987 (1985–1988)	Europe (0.99)	North America
	13	2002 (2002–2003)	Europe (1)	Asia
	14	2005 (2004–2005)	Asia (0.55)	North America
	15	2010 (2009–2010)	Europe (1)	North America
	16	2004 (2003–2005)	Europe (0.97)	Asia
	17	2013 (2013–2014)	Europe (0.99)	Asia
	18	2014 (2013–2014)	North America (0.39)	Asia
	19	2011 (2010–2011)	Europe (0.99)	North America
	20	2012 (2011–2012)	North America (0.94)	South America

a MRCA, most recent common ancestor; HPD, highest posterior density. Event numbers correspond to the numbers in Fig. 1 and Fig. S3.

**FIG 2:**
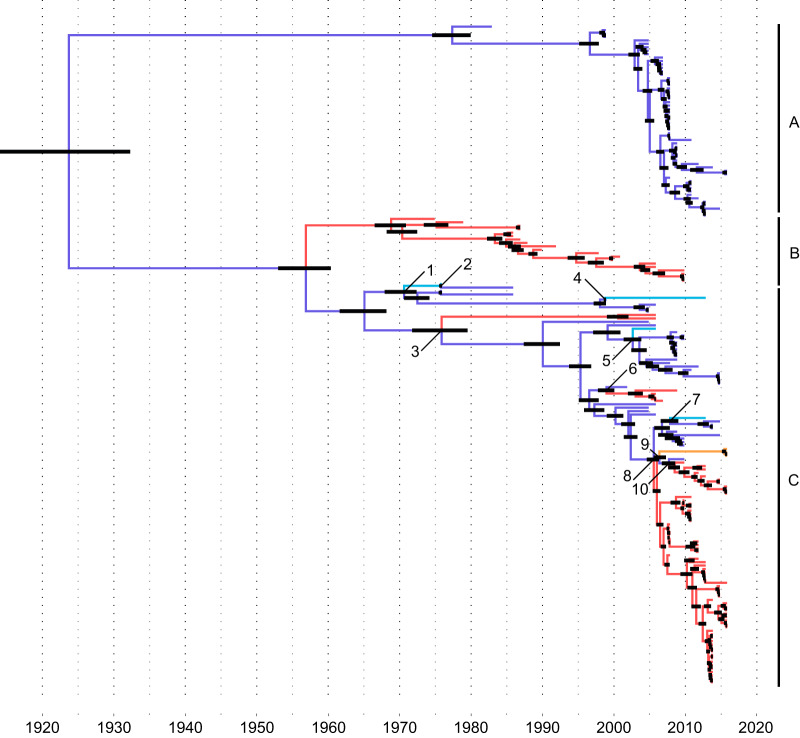
Maximum-clade-credibility (MCC) tree for influenza A virus H16 hemagglutinin subtype (*n* = 192). Branches were colored according to most probable geographic origin (red, North America; orange, South America; dark blue, Europe; light blue, Asia; green, Oceania; gray, not identified). Black node bars represent the 95% highest posterior densities for times to the common ancestors. Numbers highlight intercontinental gene flow events as detailed in Table 2 and Fig. S6. Virus strain names and posterior probabilities are presented in Fig. S5.

The H16 HA was structured in at least two major genetic lineages (Fig. 2; also, see Fig. S4 and S5). The maximum-clade-credibility (MCC) tree was structured in three main clades (A to C) (see Fig. S5), while the maximum-likelihood (ML) tree provided support for only two main genetic clades (A and B/C merged) (see Fig. S4). The tMRCA of the H16 HA gene was dated to 1924 (1914–1932). Clade A included only viruses from Europe and was dated to 1977 (1975–1980); clade B included only viruses from North America, with a tMRCA estimated at 1969 (1967–1971). Our analyses supported the idea that the geographic origins of clades A and B were Europe and North America, respectively (posterior probabilities for the geographic origin of the MRCA, 0.99 for clade A and 1 for clade B). The tMRCA of clade C was estimated to be 1965 (1962–1968). Clade C may have arisen in Europe (posterior probability for the geographic origin of the MRCA, 0.87) and consisted of viruses of mixed origin, i.e., Europe, Asia, and North America.

Since the first isolation of an H16 IAV from a black-legged kittiwake (Rissa tridactyla) in 1975, ten intercontinental gene flow events were identified for viruses of clade C (indicated by the numerals 1 to 10 in Fig. 2 and Table 2 and in Fig. S6). As for the H13 subtype, strong support for gene flow between Europe and North America was found, in particular from northwestern European countries (Denmark) to northeastern America (Delaware, New Hampshire, and Quebec) and from Iceland to Newfoundland (events 6 and 10). Evidence for intercontinental gene flow among North American H16 IAVs occurred among eastern and western North American isolates (events 3, 6, 8, and 10). In particular, intercontinental gene flow 8 seems to have been maintained in North America after its initial introduction in 2006 (2005–2006) for at least 10 years and may have replaced clade B of H16 HA (Fig. 2).

**TABLE 2:** Intercontinental gene flow events for influenza A virus H16 hemagglutinin^*a*^

H16 clade	Event	Time of MRCA ± 95% HPD	Geographic origin of the MRCA (posterior density)	Location of introduction
C	1	1971 (1968–1972)	Europe (0.97)	Asia
	2	1976 (1976–1976)	Asia (0.71)	Europe
	3	1976 (1972–1980)	Europe (0.86)	North America
	4	1999 (1999–1999)	Europe (1)	Asia
	5	2003 (2002–2004)	Europe (1)	Asia
	6	1999 (1998–2000)	Europe (0.99)	North America
	7	2008 (2007–2009)	Europe (0.99)	Asia
	8	2006 (2005–2006)	Europe (0.97)	North America
	9	2006 (2006–2007)	North America (0.55)	South America
	10	2008 (2007–2009)	Europe (0.63)	North America

a MRCA, most recent common ancestor; HPD, highest posterior density. Event numbers correspond to the numbers in Fig. 2 and Fig. S6.

High rates of nucleotide substitution obtained for the H13 HA genetic lineages were consistent with those previously reported for H4, H6, and H7 subtypes circulating in wild ducks (Table 3) (15, 38). However, the nucleotide substitution rate of clade B—which consists exclusively of North American IAV—was lower than mean rates and HPD obtained for the other two H13 clades. The mean *dN*/*dS* (nonsynonymous substitutions/synonymous substitutions) ratios obtained for the three H13 genetic clades were comparable to those previously reported for other subtypes, which suggests that the HA was under strong purifying selection (Table 3). Nonetheless, a slightly higher *dN*/*dS* ratio obtained for clade B and C compared to other lineages suggests that they may be subjected to a more neutral selection. The mean nucleotide substitution and *dN*/*dS* ratios for the H16 gene were also consistent with H13 HA as well as with H4, H6, and H7 subtypes from wild ducks. However, H16 clade C (European mixed), which consisted of viruses of a geographically more mixed origin, had slightly lower nucleotide substitution rates and higher *dN*/*dS* ratios than clade A (European) and clade B (North American) (Table 3).

**TABLE 3:** Molecular evolution of the HA gene of influenza A virus subtypes H13 and H16

Genetic lineage	*N*^*a*^	Time period (yrs)	Substitution rate^*b*^		Mean *dN*/*dS*
			Mean	95% HPD	
H13	338	40	3.8	3.6–4.1	0.13
H13 A	54	39	3.8	2.3–4.9	0.09
H13 B	76	39	0.8	0.6–1.0	0.18
H13 C	208	37	5.5	5.0–6.0	0.16
H16	192	41	3.1	2.8–3.4	0.09
H16 A	56	33	4.5	3.9–5.2	0.10
H16 B	19	35	4.6	3.9–5.2	0.06
H16 C	117	40	1.5	1.2–1.8	0.11

a Number of nucleotide sequences included in the analysis.

b Values are substitutions (10^−3^) per site per year. HPD, highest posterior density.

### Antigenic diversity between H13 and H16 LPAIVs.

**FIG 3:**
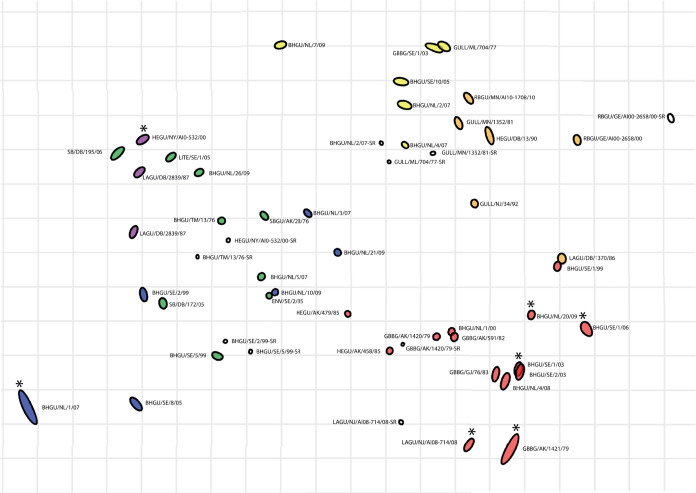
Antigenic map of H13 and H16 influenza A viruses (*n* = 44). Different subtypes and genetic clades are indicated with colors (yellow, H13 clade A; orange, H13 clade B; red, H13 clade C; blue, H16 clade A; purple, H16 clade B; green, H16 clade C). White circles indicate the antisera (*n* = 10). Virus strain names are abbreviated; the full names can be found in Table 4. Asterisks indicate antigens BHGU/NL/20/09, BHGU/SE/1/06, BHGU/SE/1/03, GBBG/AK/1421/79, BHGU/NL/1/07, HEGU/NY/AI00-532/00, and LAGU/NJ/AI08-0714/08, which had only two numerical HI titers to the tested sera, and hence, their placement in the map is not robust. In this map, the distance between the points represents antigenic distance as measured by the hemagglutination inhibition (HI) assay, in which the distances between antigens and antisera are inversely related to the log_2_ HI titer. Each square in the grid of the antigenic map equals a 2-fold difference in the HI assay.

As expected from two different HA subtypes, the H13 and H16 viruses formed two separate antigenic variants. The H13 and H16 viruses were generally well separated, forming groups on opposite sides of the antigenic map (Fig. 3; Table 4). A total of nine amino acid positions within or near the receptor binding site of the HA were identified that differed consistently between H13 and H16 viruses (based on alignments of 338 H13 and 192 H16 HA indicated in Table 5); of those, amino acid position 145 was located in the 130 loop, 200 and 208 were in the 190 helix, and 231 and 233 were in the 220 loop of the receptor binding site of the HA (HA numbering based on references 28 and 29). Of those, amino acid position 233 was listed previously as being involved in differences in receptor-binding site between HAs originating from Laridae and Anatidae (30). Additionally, the amino acid at position 196 differed between H13 (valine [V]) and H16 (aspartic acid [D]) viruses; this position may contribute to receptor binding specificity as identified previously based on crystal structures of H5 and H13 LPAIV (31). Due to nonspecific cross-reactivity, two H13 viruses (i.e., HEGU/AK/458/85 and HEGU/AK/479/85) had unexpected high titers against H16 antisera (Table 4); they were therefore positioned in the center of the map and served to pull H13 and H16 together.

**TABLE 4:** Hemagglutinin inhibition data of H13 and H16 influenza A viruses (*n* = 44)

Subtype and clade	Virus isolate	Subtype	Virus abbreviation	Antisera^*a*^:									
				H13						H16			
				A	A	B	B	C	C	A	B	C	C
				BHGU/NL/2/07	GULL/MD/704/77	GULL/MN/1352/81	RBGU/GE/AI00-2658/00	GBBG/AK/1420/79	LAGU/NJ/AI08-714/08	BHGU/SE/2/99	HEGU/NY/AI0-532/00	BHGU/SE/5/99	BHGU/TM/13/76
H13/A	A/Black-headed gull/Netherlands/2/07	H13N6	BHGU/NL/2/07	**320**	280	80	<10	20	<10	<10	<10	<10	25
	A/Black-headed gull/Netherlands/4/07	H13N6	BHGU/NL/4/07	1,280	400	320	<10	35	<10	<10	<10	10	40
	A/Black-headed gull/Netherlands/7/09	H13N2	BHGU/NL/7/09	10	160	<10	<10	<10	<10	10	<10	<10	15
	A/Black-headed gull/Sweden/10/05	H13N6	BHGU/SE/10/05	240	320	40	<10	10	<10	<10	<10	<10	15
	A/Great-black headed gull/Sweden/1/03	H13N6	GBBG/SE/1/03	80	240	20	<10	<10	<10	<10	<10	<10	<10
	A/gull/MD/704/77	H13N6	GULL/MD/704/77	40	**240**	20	<10	<20	<10	<10	<10	<10	<10
H13/B	A/gull/MN/1352/81	H13N6	GULL/MN/1352/81	120	160	**320**	<10	20	<10	<10	<10	<10	<10
	A/gull/NJ/34/92	H13N6	GULL/NJ/34/92	80	240	80	<10	240	<10	<10	<10	<10	<10
	A/Herring gull/DB/13/90	H13N2	HEGU/DB/13/90	40	140	140	10	25	<10	<10	<10	<10	<10
	A/Laughing gull/DB/1370/86	H13N2	LAGU/DB/1370/86	10	40	<10	10	40	<10	<10	<10	<10	<10
	A/ring-billed gull/GE/AI00-2658/00	H13N6	RBGU/GE/AI00-2658/00	10	60	40	**640**	15	<10	<10	<10	<10	<10
	A/ring-billed gull/MN/AI10-1708/10	H13N6	RBGU/MN/AI10-1708/10	80	200	120	10	10	<10	<10	<10	<10	<10
H13/C	A/Black-headed gull/Netherlands/1/00	H13N8	BHGU/NL/1/00	35	<10	<10	<10	1,280	120	<10	30	<10	30
	A/Black-headed gull/Netherlands/20/09	H13N2	BHGU/NL/20/09	<10	<10	<10	<10	280	<10	<10	<10	<10	35
	A/Black-headed gull/Netherlands/4/08	H13N8	BHGU/NL/4/08	<10	<10	<10	<10	140	80	<10	<10	<10	25
	A/Black-headed gull/Sweden/1/03	H13N8	BHGU/SE/1/03	<10	<10	<10	<10	560	40	<10	<10	<10	<10
	A/Black-headed gull/Sweden/1/06	H13N8	BHGU/SE/1/06	<10	<10	<10	<10	120	<10	<10	<10	<10	<10
	A/Black-headed gull/Sweden/1/99	H13N6	BHGU/SE/1/99	10	<10	10	30	160	<10	<10	<10	<10	10
	A/Black-headed gull/Sweden/2/03	H13N8	BHGU/SE/2/03	<10	<10	<10	<10	200	50	<10	<10	<10	10
	A/Great-black headed gull/AK/1420/79	H13N2	GBBG/AK/1420/79	10	35	10	<10	**2,720**	160	10	<10	35	25
	A/Great-black headed gull/AK/1421/79	H13N2	GBBG/AK/1421/79	<10	<10	<10	<10	140	80	<10	<10	<10	<10
	A/Great-black headed gull/AK/591/82	H13N2	GBBG/AK/591/82	<10	40	<10	<10	480	100	<10	<10	40	80
	A/Great-black headed gull/GJ/76/83	H13N2	GBBG/GJ/76/83	<10	<10	<10	<10	320	80	<10	<10	<10	30
	A/Herring gull/AK/458/85	H13N6	HEGU/AK/458/85	30	20	<10	<10	1,920	480	70	<10	80	80
	A/Herring gull/AK/479/85	H13N6	HEGU/AK/479/85	140	35	10	<10	1,920	640	280	120	280	120
	A/Laughing gull/NJ/AI08-714/08	H13N9	LAGU/NJ/AI08-714/08	<10	<10	<10	<10	320	**560**	<10	<10	<10	<10
H16/A	A/Black-headed gull/Netherlands/5/07	H16N3	BHGU/NL/5/07	35	25	<10	<10	140	<10	960	160	320	640
	A/Black-headed gull/Netherlands/1/07	H16N3	BHGU/NL/1/07	<10	<10	<10	<10	<10	<10	80	<10	<10	40
	A/Black-headed gull/Netherlands/10/09	H16N3	BHGU/NL/10/09	20	80	<10	<10	280	15	1,280	160	640	640
	A/Black-headed gull/Netherlands/21/09	H16N3	BHGU/NL/21/09	70	200	20	<10	240	<10	480	<10	240	280
	A/Black-headed gull/Netherlands/3/07	H16N3	BHGU/NL/3/07	100	90	20	<10	100	<10	120	140	60	120
	A/Black-headed gull/Sweden/2/99	H16N3	BHGU/SE/2/99	10	<10	<10	<10	10	<10	**960**	80	35	380
	A/Black-headed gull/Sweden/8/05	H16N3	BHGU/SE/8/05	<10	<10	<10	<10	10	<10	1280	<10	30	140
H16/B	A/Herring gull/DB/2617/87	H16N3	HEGU/DB/2617/87	<10	<10	<10	<10	<10	<10	<10	120	20	1,600
	A/Herring gull/NY/AI0-532/00	H16N3	HEGU/NY/AI0-532/00	<10	<10	<10	<10	<10	<10	<10	**320**	<10	320
	A/Laughing gull/DB/2839/87	H16N3	LAGU/DB/2839/87	<10	<10	<10	<10	<10	<10	160	80	20	1920
H16/C	A/Black-headed gull/Netherlands/26/09	H16N3	BHGU/NL/26/09	10	25	<10	<10	20	<10	30	80	20	1280
	A/Black-headed gull/Sweden/5/99	H16N3	BHGU/SE/5/99	10	<10	<10	<10	70	<10	560	30	**1,600**	400
	A/Black-headed gull/TM/13/76	H16N3	BHGU/TM/13/76	25	30	<10	<10	27.5	<10	50	320	100	**4,800**
	A/environment/Sweden/2/05	H16N3	ENV/SE/2/05	20	30	10	<10	140	30	960	320	1,280	640
	A/Little tern/Sweden/1/05	H16N3	LITE/SE/1/05	<10	15	<10	<10	15	<10	10	30	20	1,280
	A/shorebird/DB/172/05	H16N3	SB/DB/172/05	<10	<10	<10	<10	30	<10	240	60	200	1,280
	A/shorebird/DB/195/06	H16N3	SB/DB/195/06	<10	<10	<10	<10	<10	<10	<10	30	20	560
	A/Slender-billed gull/AK/28/76	H16N3	SBGU/AK/28/76	20	140	10	<10	50	<10	80	160	100	1,280

a Boldface indicates homologous titers.

**TABLE 5:** Amino acid differences within or near the receptor binding site of the HA protein among H13 and H16 subtypes and clades^*a*^

Clade	Amino acid(s) at position:															
	139	142	145	149	166	176	177	196	198	200	208	217	218	224	231	233
H13 A	D	A, T, S	A	D, E, N, S	K, Q	K	T	V, L	V	E	S, G	K	S, L	K	P	Y
H13 B	D	A, T, S	A	D, N, S	K, R	G, R	T	V, I	T, A	E	S, G	S, R, N, H	S, L	K, N	P, L	Y, Q
H13 C	D	V, A	A	DEL, R	K, R, S	G, R	T, A, V	V, I	T, A, E	E	D, N, S	S, R, G	S, T	N, T, K	P	Y
H16 A	E	T	S	DEL	L	G	E	D	E	T	K	K	E	E	I	D
H16 B	D	V	S	DEL	DEL	G	D	D	E, ?	T, V	K	K, E	E	E	I	D, E, N
H16 C	D	V, A	S	DEL	K, DEL	G	E, D	D	E	T	K	K	E	E	I, V	D, N

a Based on the HA gene of H13 (*n* = 338) and H16 (*n* = 192) LPAIVs, including the 130 loop (positions 136 to 147 according to reference 29), the 190 helix (200 to 208), and the 220 loop (230 to 240). DEL, deletion of the amino acid.

### Antigenic diversity among H13 LPAIVs.

The representative H13 viruses formed at least two different antigenic variants (Fig. 3; Table 4). The viruses of H13 clades A and B were genetically distinct (Fig. 1) but were antigenically similar (Fig. 3), based on the H13 clade A antisera cross-reacting with H13 clade B viruses and vice versa. In contrast, H13 clade C viruses reacted poorly—if at all—with antisera that were raised against clade A and B viruses, and, conversely, antisera against clade C viruses rarely reacted with substantial titers with viruses of clade A and B. Thus, H13 clade A/B and H13 clade C viruses formed two different antigenic variants. The antigenic diversity of H13 clades A and B combined is about the same as the antigenic diversity of the H13 clade C. One H13 clade B virus, i.e., LAGU/DB/1370/86, could not be placed well in the map due to hemagglutination inhibition (HI) titers of 40 or lower (Table 4).

To gain insight into the molecular basis of the antigenic variation between H13 clades A/B and C, amino acids that differed consistently among the different clades of H13 viruses were identified (based on the alignment of 338 H13 [Table 5]). A total of four amino acid positions within or near the receptor binding site of the HA were identified that differed consistently for clades A, B, and/or C. Of those, amino acids at positions 149 and 254 differed consistently between clades A/B and C. Viruses belonging to clade C—except a single virus from South America that had an arginine (R) at position 149—had a deletion at position 149 (previously identified using a smaller data set as position 154 [12]), in contrast to viruses of clade A or B, which had an aspartic acid (D), glutamic acid (E), asparagine (N), or serine (S) at this position. The correlation between the antigenic distance of H13 representative viruses from A/gull/MD/704/1977 (H13N6) (clade A)—the first detected H13 virus—and the number of HA1 amino acid substitutions from A/gull/MD/704/1977 was 0.87 and was statistically significant (*P* < 0.0001; Pearson correlation).

### Antigenic diversity among H16 LPAIVs.

The representative H16 viruses formed at least one antigenic variant (Fig. 3 and Table 4). The genetically distinct H16 clades A, B, and C did not form separate antigenic clusters in the map, which reflects the raw HI data, as there are no patterns for any of the four H16 antisera tested that correspond to the genetic lineages. The antigenic diversity of the H16 viruses is within eight antigenic units, with BHGU/NL/1/07 being on the edge of this antigenic space (i.e., low titers to all sera). The antigenic diversity of H16 clade A/B/C is about the same as the antigenic diversity of the H13 clades A and B combined and similar to the antigenic diversity of the H13 clade C.

Though clades A, B, and C did not form separate antigenic clusters in our analysis, amino acids that differed consistently among the different clades of H16 viruses were identified (based on the alignment of 192 H16 HA [Table 5]). A total of three amino acid positions within or near the receptor binding site of the HA were identified that differed consistently among the three H16 clades and were not associated with antigenic variation. The correlation between the antigenic distance of the representative viruses from A/Black-headed gull/TM/13/76 (H16N3) (clade C)—one of the first detected H16 viruses—and the number of HA1 amino acid substitutions from A/Black-headed gull/TM/13/76 was 0.67 and was statistically significant (*P* = 0.003; Pearson correlation).

## DISCUSSION

We investigated the evolutionary history and intercontinental gene flow based on the hemagglutinin (HA) gene of H13 and H16 LPAIV and selected representative viruses from genetically distinct lineages to determine their antigenic properties by HI assays. H13 formed at least three distinct genetic clades as suggested previously based on smaller data sets (9, 32–35), while H16 formed at least two distinct genetic clades. Twenty and ten events of intercontinental gene flow were identified for H13 and for H16 viruses, respectively. At least two antigenic variants of H13 and at least one antigenic variant of H16 were identified. The presence of different antigenic variants among viruses of a single LPAIV subtype is in contrast to previous findings based on antigenic characterization of LPAIV H3 (26) and implies that antigenic variation within LPAIV subtypes occurs.

The frequency of intercontinental gene flow of the HA gene of H13 and H16 viruses was similar to that of the HA gene of H6 viruses but lower than that of internal genes (2, 27, 36, 37). Previously, intercontinental gene flow was described extensively for the H6 HA genes, while no intercontinental gene flow was detected for the H4 and H7 subtypes (15, 38). For the H6 subtype, gene flow has been described to have occurred ten times with four established genes during a period of 31 years (1975 to 2006) (15). Also, evidence for intercontinental gene flow among North American H13 and H16 genes occurred among eastern and western North American LPAIVs, in contrast to eastern North American LPAIVs only, as reported previously (39). Given the relatively high number of intercontinental flow events of IAV internal genes associated with shorebirds and gulls (2, 27, 36, 37), one might have expected a higher gene flow of gull-associated H13 and H16 HA genes, compared to, e.g., H6. However, a higher intercontinental gene flow was apparent only with H13 (i.e., 20 events during a period of 35 years). This may suggest one or more of the following: (i) H13 has a broader host range, host population size, and/or host distribution than H16; (ii) local H13-specific herd immunity is lower than H16-specific herd immunity and therefore less limiting of establishment opportunities in host populations of H13; (iii) H13 has higher environmental survival than H16; and (iv) introduced H13 HA genes may be less affected by strong subtype-dependent competition with endemic HA genes (e.g., with respect to linkage to NS1 and NP, as these contain most gull-specific features [33]) than introduced H16 genes. Interestingly, no H13 or H16 gene flow from Asia to Europe was described, which is in contrast to, e.g., H5 HPAIVs that have been introduced from Asia to Europe several times (40, 41). The relatively low frequency of detection of intercontinental gene flow of H13 or H16 genes out of North America and in particular Asia, relative to Europe, may be due to a bias in IAV surveillance and sequencing (i.e., the number of available IAV sequences from gulls isolated in Europe is higher than those from North America and in particular Asia).

Antigenic diversity of LPAIVs depends partially on the host population size and structure. In this study, H13 and H16 LPAIVs formed at least three and two distinct genetic clades, respectively, that did not correspond or only partially corresponded with antigenic clusters. The H16 genetic clades did not form antigenic clusters, suggesting that clade-defining mutations were not in critical epitopes. In contrast, the H13 genetic clades partially corresponded with the antigenic variation of H13 LPAIV, suggesting that some of the clade-defining mutations were in critical epitopes. Also, given that the H13 antigenic space is larger than the antigenic space covered by H16 viruses, the host population of H13 may be larger and more widely distributed than the host population of H16 LPAIV, facilitating the circulation of more than one antigenic variant of a single LPAIV subtype. Strong genetic and antigenic divergence between two cocirculating lineages could be the product of a very large host meta-population size and relatively low cross-species transmission rate (42). Globally, viruses of the H13 subtype seem to be more common than viruses of the H16 subtype (2, 4), which is consistent with the finding that H13 LPAIV consists of multiple antigenic variants. Besides increased host population size and host distribution, prolonged virus survival may shape LPAIV epidemiology and evolution. Antigenic diversity within H13 LPAIV may be shaped by amino acid substitutions near the receptor binding site of the HA protein. In this study, we found evidence that amino acids or deletions at positions 149 and 254 of the HA protein may be involved in antigenic diversity among H13 strains. In addition, position 149 could be involved in H16 LPAIV antigenic diversity, as all H16 viruses had a deletion at this position and H16 clades A, B, and C were antigenically similar.

Cocirculating and newly introduced H13 or H16 LPAIV can be either antigenically similar or antigenically different. In the Northern hemisphere, H13 and H16 IAV subtypes circulate most extensively in breeding colonies in hatch-year birds at the end of summer and early fall (5–7). In black-headed gulls (which in Europe are one of the main hosts for H13 and H16 LPAIVs), infection with H13 or H16 results in strong protection against reinfection with the same virus; however, susceptibility to infection with the other subtype or with another strain of the same subtype is unknown (43, 44). Our findings support the independent long-term maintenance and cocirculation of at least two genetically distinct lineages of H13 and of H16 in Eurasia. This pattern is similar to the one that has been described for the H3 IAV subtype in ducks in North America (42). Our analysis showed that these genetically distinct cocirculating lineages may belong to the same antigenic variant. Here, we found evidence that genetically distinct cocirculating H13 or H16 LPAIV in a black-headed gull breeding colony site in the Netherlands may be either antigenically different (e.g., H13 clade A virus A/BHGU/NL/7/2009 [H13N2] and H13 clade C virus A/BHGU/NL/20/2009 [H13N2]) or antigenically similar (e.g., H16 clade A viruses A/BHGU/NL/10/2009 [H16N3] and A/BHGU/NL/21/2009 [H16N3] and H16 clade C virus A/BHGU/NL/26/2009 [H16N3]). Similarly, intercontinental gene flow occurred with HA genes that were antigenically similar to local circulating viruses (i.e., H16 clade C viruses that were genetically closely related to SB/DB/172/06 and SB/DB/195/06 versus local circulating H16 clade B viruses) and HA genes that were antigenically different from local circulating viruses (i.e., H13 clade C viruses genetically closely related to LAGU/NJ/AI08-0714/08 versus local circulating H13 clade B viruses.

Antigenic variation within an LPAIV subtype at the clade level (i.e., H13 clades A and B combined versus H13 clade C) was described here, yet less is known about antigenic variation within genetic clades of H13, H16, or other LPAIV subtypes. For H13, genetic diversity within clades seemed stable—i.e., viruses of clade A, B, or C collected over 3 decades were antigenically closely related—suggesting no major genetic differences; this is in contrast to the few mutations needed for antigenic change in seasonal human IAV. Similarly, a study on antigenic variation of H3 LPAIV isolated in North America suggested that genetically diverse viruses were antigenically stable (26). Major antigenic changes in seasonal human IAV were due to amino acid substitutions immediately adjacent to the receptor binding site (18); this could potentially also explain antigenic variation between antigenically different viruses of H13 clade A/B and clade C (i.e., amino acid position 149 of the HA). Future work on antigenic variation of LPAIV should include within-clade genetic and antigenic variation.

## MATERIALS AND METHODS

### Viruses.

The HA sequences of H13 (*n* = 64) and H16 (*n* = 20) viruses isolated from wild birds in North America (*n* = 39 and *n* = 5, respectively) and Europe (*n* = 25 and *n* = 15, respectively) between 1976 and 2010 were determined at the University of Minnesota (Saint Paul, MN) and at the Department of Viroscience of the Erasmus Medical Center (Rotterdam, the Netherlands). Details on virus isolates, including GenBank accession numbers, are summarized in Tables S2 and S3; details related to the Sanger sequencing method are available upon request. The HA sequences were supplemented with full-length nucleotide sequences of the HA gene of H13 and H16 viruses isolated from wild birds between 1975 and 2017 and downloaded from GenBank (https://www.ncbi.nlm.nih.gov). The full data set included sequences of H13 (*n* = 519) and H16 (*n* = 276) HA genes and was biased toward virus strains collected since 2000 due to increased surveillance and sequencing since 2000.

Of this full data set, viruses representing the genetically distinct clades were selected (*n* = 44; H13 clades A, B, and C and H16 clades A, B, and C; see Results for clade definition) to investigate the antigenic diversity of H13 and H16 viruses. Of those viruses, viruses that were genetically most divergent were selected (*n* = 10) to generate ferret antisera (Table 6). The antigenic properties of all representative viruses (*n* = 44) were analyzed in hemagglutination inhibition (HI) assays using a panel of ten ferret antisera.

**TABLE 6:** Representative viruses selected to generate ferret antisera used to map the antigenic diversity of H13 and H16 influenza A viruses

Subtype	Clade	Virus strain name
H13	A	A/gull/Maryland/704/1977 (H13N6)
	A	A/Black-headed gull/Netherlands/2/2007 (H13N6)
	B	A/Ring-billed gull/Georgia/AI00-2658/2000 (H13N6)
	B	A/gull/Minnesota/1352/1981 (H13N6)
	C	A/Laughing gull/New Jersey/AI08-0714/2008 (H13N9)
	C	A/Great black-headed gull/Astrakhan/1420/1979 (H13N2)
H16	A	A/Black-headed gull/Sweden/2/1999 (H16N3)
	B	A/Herring gull/New York/AI00-532/2000 (H16N3)
	C	A/Black-headed gull/Turkmenistan/13/1976 (H16N3)
	C	A/Black-headed gull/Sweden/5/1999 (H16N3)

### Genetic analyses.

The nucleotide sequences of the coding region of H13 and H16 HA were aligned with the program CLC 8.0 (CLC bio, Aarhus, Denmark). Neighbor-joining trees were then generated, with 1,000 bootstraps, in order to assess the overall genetic structure of the H13 (*n* = 519) and H16 (*n* = 276) HA sequences. To lower the bias in species and geography (e.g., black-headed gulls [Chroicocephalus ridibundus] from the Netherlands and glaucous-winged gulls [Larus glaucescens] from Alaska), duplicate sequences (i.e., identical sequences of the same host species, location, and date) were identified with mothur 1.39.5 (45) and removed, resulting in final alignments of H13 (*n* = 338) and H16 (*n* = 192) HA. To identify the genetic structure of H13 and H16 virus subtypes, maximum-likelihood trees with 1,000 bootstraps were generated with the software PhyML 3.1 (46). The general time-reversible (GTR) evolutionary model, an estimation of the proportion of invariable sites (I) and of the nucleotide heterogeneity of substitution rate (α), was used as selected by Model Generator 0.85 (47). To investigate the evolutionary history of H13 and H16 virus subtypes, Bayesian Markov chain Monte Carlo coalescent analyses were performed. The temporal structure of the data set was assessed with the program TempEst 1.5.3 (48). Both data sets showed a positive correlation between genetic divergence and sampling time and appear to be suitable for phylogenetic molecular clock analyses. Time to the most recent common ancestors (MRCA) as well as geographic ancestral states (i.e., continent) and their associated posterior probabilities were obtained based on the method described by Lemey et al. with the program BEAST 1.10.1 (49, 50). A strict molecular clock model was selected, as relaxed clock models (uncorrelated exponential and uncorrelated log-normal) resulted in low effective sample sizes (< 200) in spite of high chain length (>200 million states). In all simulations, a Bayesian skyline coalescent tree prior (51) was selected. The Shapiro-Rambaut-Drummond-2006 nucleotide substitution model was selected (52), and it has been used in population dynamic studies of other IAV subtypes (15, 38, 42, 53). Overall, a method similar to that in previous studies on IAV evolutionary dynamics of subtypes H4, H6, and H7 (15, 38, 54) was used. Analyses were performed with two independent chain lengths of 100 million generations sampled every 1,000 iterations; the first 10% of trees were discarded as burn-in. Substitutions rates based on independent analyses of the major H13 and H16 clades were obtained using the program BEAST 1.10.1. Rates of nonsynonymous substitutions (*dN*) and synonymous substitutions (*dS*) were obtained using the single likelihood ancestor counting method implemented in HyPhy (55). Computations were performed with the Datamonkey Web server (56, 57).

### Antisera.

Postinfection antisera were prepared upon nasal inoculation of ferrets (>1 year of age; male; two ferrets per virus) with virus (cultured on embryonated chicken eggs; each ferret received 106 to 107 median egg infectious doses [EID_50_]/100 μl) and blood collection by exsanguination 14 days later. An overview of antisera used in this study is provided in Table 6. Antisera were pretreated overnight at 37°C with receptor-destroying enzyme (Vibrio cholerae neuraminidase), followed by inactivation for 1 h at 56°C before use in HI assays.

### Antigenic analyses.

HI assays were performed according to standard procedures (58). The HI titer is expressed as the reciprocal value of the highest serum dilution that completely inhibited hemagglutination. To investigate antigenic variation among and within H13 and H16 viruses, antigenic cartography methods were used as described previously (19). Briefly, antigenic cartography is a method to analyze and visualize HI assay data. The titers in an HI table can be thought of as specifying target distances between antigens and antisera. In an antigenic map, the distance between antigen point A and antiserum point S corresponds to the difference between the log_2_ value of the maximum observed titer to antiserum S from any antigen and the titer of antigen A to antiserum S. Modified multidimensional scaling methods are used to arrange the antiserum and antigen points in an antigenic map to best satisfy the target distances specified by the HI data (18). Because antigens are tested against multiple antisera and antisera are tested against multiple antigens, many measurements can be used to determine the position of the antigens and antisera in an antigenic map, thus improving the resolution of the HI data.

### Ethics statement.

This study was approved by the independent animal experimentation ethical review committee Stichting DEC consult (Erasmus MC permits 122-98-01, 122-08-04, and 15–340-03) and was performed under animal biosafety level 2 (ABSL2) conditions. Animal welfare was monitored daily, and all animal handling was performed under light anesthesia (ketamine) to minimize animal discomfort.

### Data availability.

Sequences are available in GenBank under accession numbers KF612922 to KF612965, KR087564, KR087572, KR087577 to KR087595, KR087597 to KR087601, KR087604 to KR087615, MK027211, and MK027212.

## Supplementary Material

Supplemental file 1
