# Supplementary material for: Phylogeography and Antigenic Diversity of Low-Pathogenic Avian Influenza H13 and H16 Viruses
Source: J Virol. 2020 Jun 16;94(13):e00537-20. doi: 10.1128/JVI.00537-20 (PMC7307148; doi:10.1128/JVI.00537-20)
Supplement: Supplemental file 1 [file JVI.00537-20-s0001.pdf]

Supplementary Material of

"Phylogeography and antigenic diversity of low pathogenic avian influenza H13 and H16 viruses"

Figure S1. Maximum-Likelihood tree obtained with H13 HA sequences (n=338) and 1000 bootstraps. Virus names in bold were sequenced as part of this study. Those highlighted in red were used for the antigenic analyses. Only bootstrap values higher than 50 are indicated.

Figure S2. Maximum clade credibility tree for influenza A virus H13 hemagglutinin subtype (n=338). Posterior probabilities are reported when higher than 0.5. Virus names in bold were sequenced as part of this study. Those highlighted in red were used for the antigenic analyses. Node bars indicate 95% highest posterior density for times of the most recent common ancestors. Scale bar indicates 10 years.

Figure S3. World map indicating intercontinental gene flow of influenza A virus H13 hemagglutinin in time. Numbers highlight intercontinental gene flow events as detailed in Table 2 and Figure 1. Arrows indicate direction of gene flow. Colors indicate time interval between the most recent common ancestor (MRCA) and the detected H13 LPAIV. Continuous line: posterior probability of >0.95; dotted line: posterior probability of ≤0.95. Map from amCharts, https://www.amcharts.com/svg-maps/?map=world; CC BY-NC 4.0.

Figure S4. Maximum-Likelihood tree obtained with H16 HA sequences (n=192) and 1000 bootstraps. Virus names in bold were sequenced as part of this study. Those highlighted in red were used for the antigenic analyses. Only bootstrap values higher than 90 are indicated.

Figure S5. Maximum clade credibility tree for influenza A virus H16 hemagglutinin subtype (n=192). Posterior probabilities are reported when higher than 0.5. Virus names in bold were sequenced as part of this study. Those highlighted in red were used for the antigenic analyses. Node bars indicate 95% highest posterior density for times of the most recent common ancestors. Scale bar indicates 10 years.

Figure S6. World map indicating intercontinental gene flow of influenza A virus H16 hemagglutinin in time. Numbers highlight intercontinental gene flow events as detailed in Table 3 and Figure 2. Arrows indicate direction of gene flow. Colors indicate time interval between the most recent common ancestor (MRCA) and the detected H16 LPAIV. Continuous line: posterior probability of >0.95; dotted line: posterior probability of ≤0.95. Map from amCharts, https://www.amcharts.com/svg-maps/?map=world; CC BY-NC 4.0.

Table S1. Distribution of influenza A virus subtypes among gull species in Eurasia and America based on the Influenza Research Database (IRD, https://www.fludb.org) (d.d. 20-Dec-2019). Subtype not detected (-)

Table S2. List of H13 HA influenza A viruses (n=519) and corresponding accession number included in the study. Virus names in bold were sequenced as part of this study.

Table S3. List of H16 HA influenza A viruses (n=276) and corresponding accession numbers included in the study. Virus names in bold were sequenced as part of this study.

Figure S1

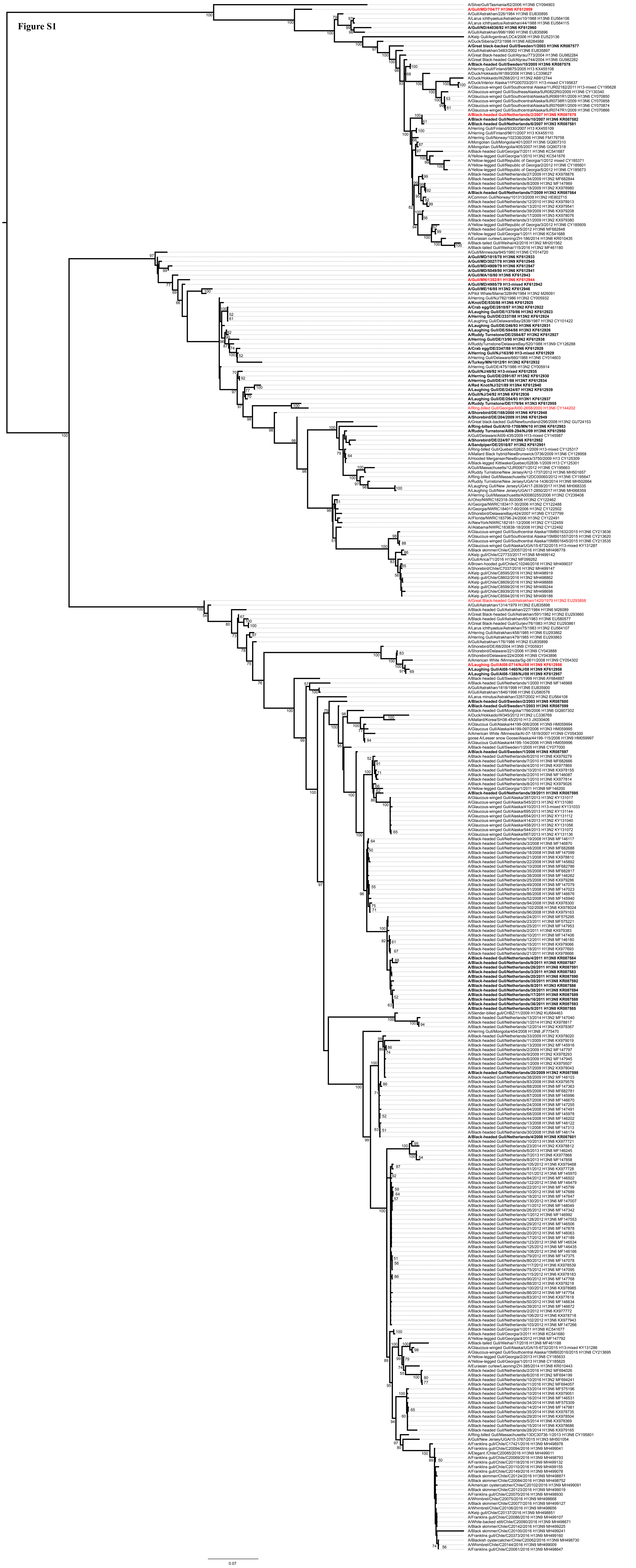

Figure S2

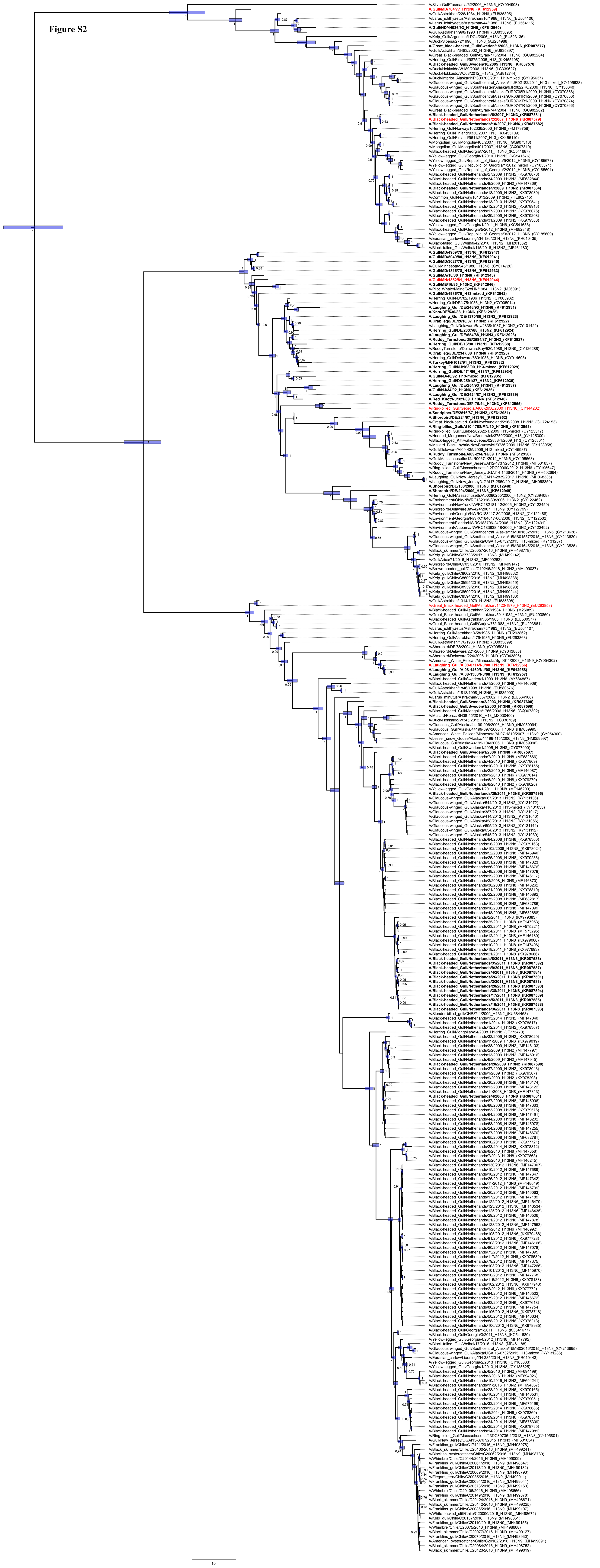

Figure S3

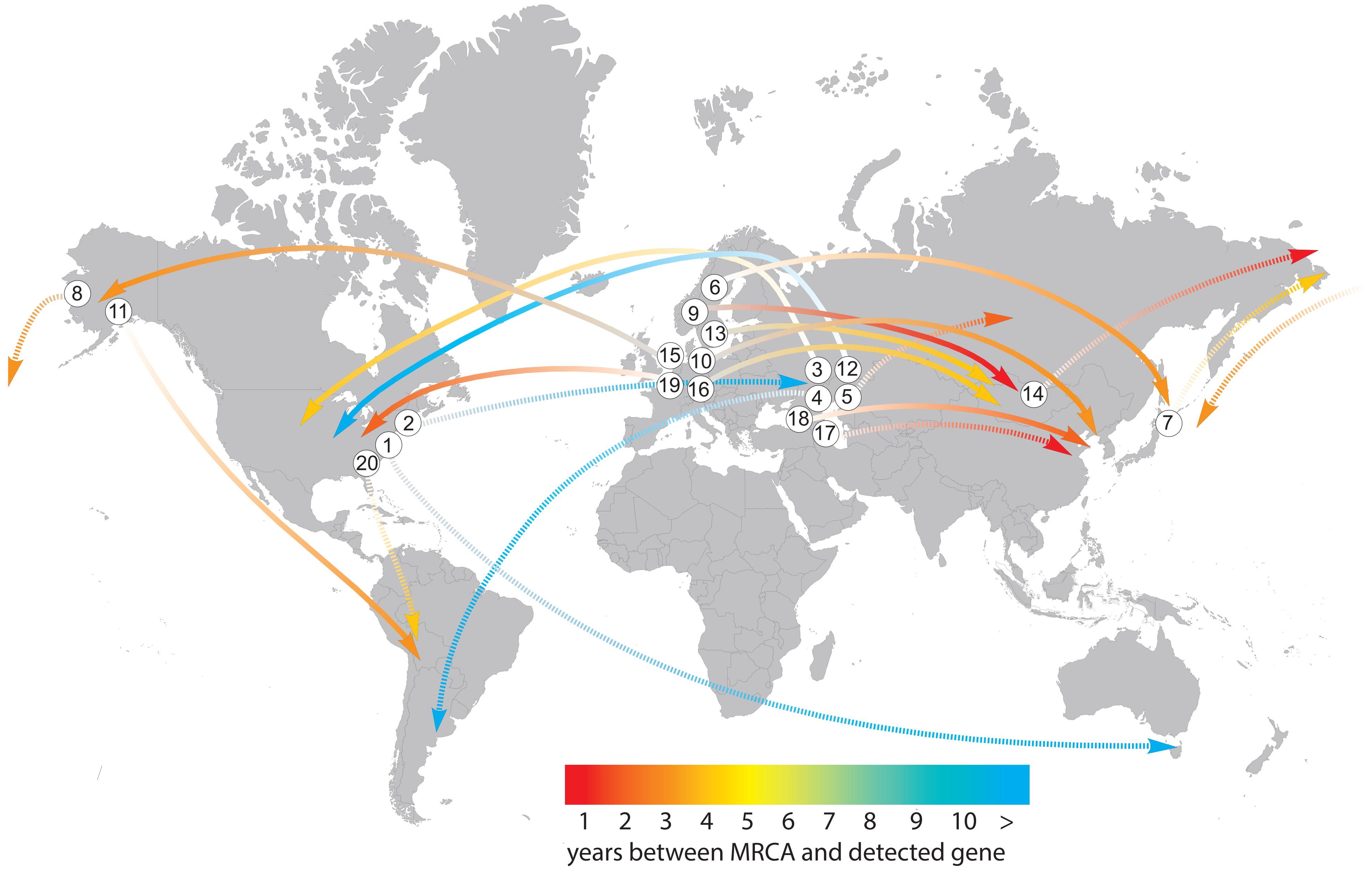
1
2
3
4
5
6
7
8
9
10
11
12
13
14
15
16
17
18
19
20
1 2 3 4 5 6 7 8 9 10 >
years between MRCA and detected gene

Figure S4

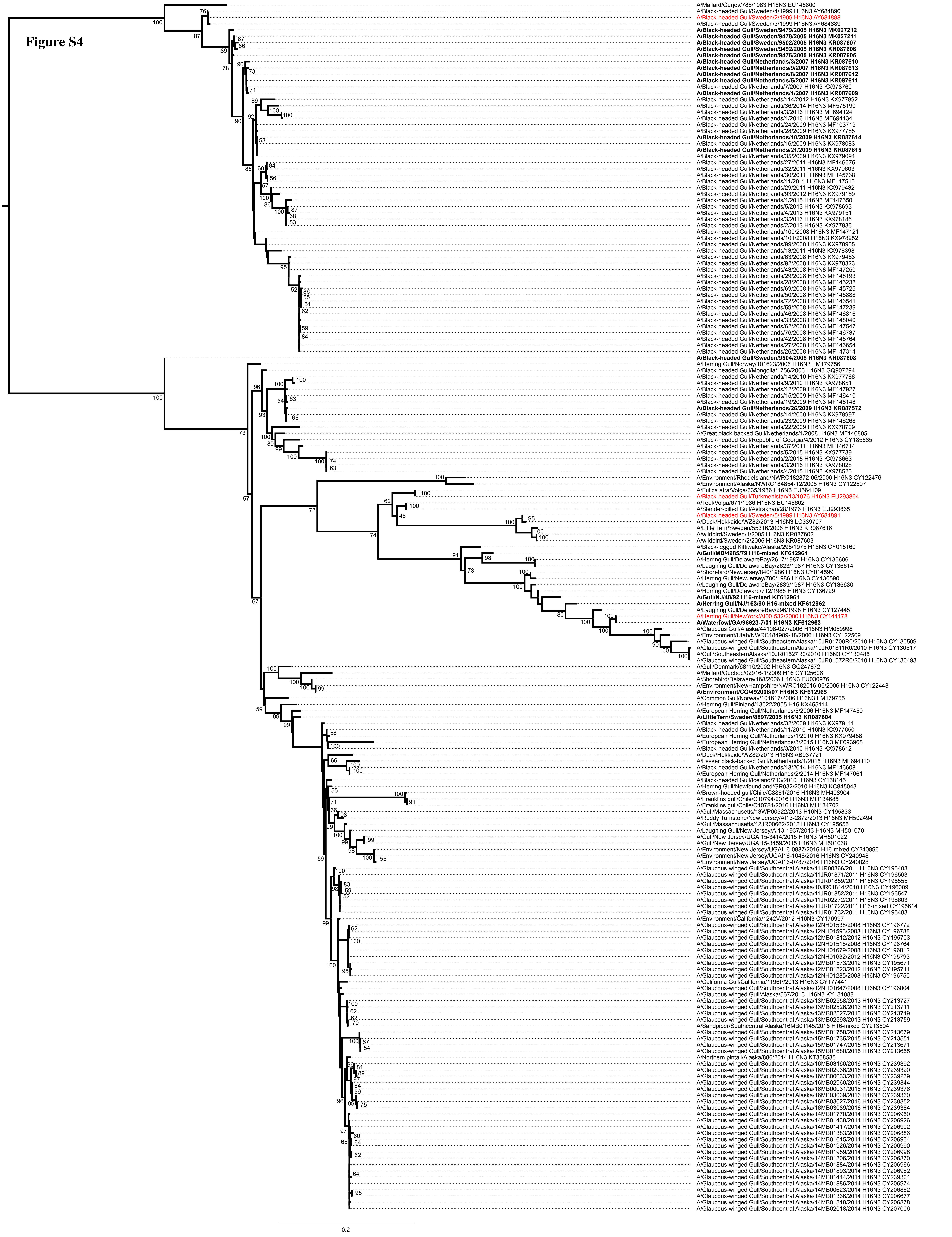

A/Mallard/Gurjev/785/1983 H16N3 EU148600
A/Black-headed Gull/Sweden/4/1999 H16N3 AY684890
A/Black-headed Gull/Sweden/2/1999 H16N3 AY684888
A/Black-headed Gull/Sweden/3/1999 H16N3 AY684889
A/Black-headed Gull/Sweden/9479/2005 H16N3 MK027212
A/Black-headed Gull/Sweden/9478/2005 H16N3 MK027211
A/Black-headed Gull/Sweden/9502/2005 H16N3 KR087607
A/Black-headed Gull/Sweden/9492/2005 H16N3 KR087606
A/Black-headed Gull/Sweden/9476/2005 H16N3 KR087605
A/Black-headed Gull/Netherlands/3/2007 H16N3 KR087610
A/Black-headed Gull/Netherlands/9/2007 H16N3 KR087613
A/Black-headed Gull/Netherlands/8/2007 H16N3 KR087612
A/Black-headed Gull/Netherlands/5/2007 H16N3 KR087611
A/Black-headed Gull/Netherlands/7/2007 H16N3 KX978760
A/Black-headed Gull/Netherlands/1/2007 H16N3 KR087609
A/Black-headed Gull/Netherlands/114/2012 H16N3 KX977892
A/Black-headed Gull/Netherlands/36/2014 H16N3 MF575190
A/Black-headed Gull/Netherlands/3/2016 H16N3 MF694124
A/Black-headed Gull/Netherlands/1/2016 H16N3 MF694134
A/Black-headed Gull/Netherlands/24/2009 H16N3 MF103719
A/Black-headed Gull/Netherlands/28/2009 H16N3 KX977785
A/Black-headed Gull/Netherlands/10/2009 H16N3 KR087614
A/Black-headed Gull/Netherlands/16/2009 H16N3 KX978083
A/Black-headed Gull/Netherlands/21/2009 H16N3 KR087615
A/Black-headed Gull/Netherlands/35/2009 H16N3 KX979094
A/Black-headed Gull/Netherlands/27/2011 H16N3 MF146675
A/Black-headed Gull/Netherlands/32/2011 H16N3 KX979603
A/Black-headed Gull/Netherlands/30/2011 H16N3 MF145738
A/Black-headed Gull/Netherlands/11/2011 H16N3 MF147513
A/Black-headed Gull/Netherlands/29/2011 H16N3 KX979432
A/Black-headed Gull/Netherlands/93/2012 H16N3 KX979159
A/Black-headed Gull/Netherlands/1/2015 H16N3 MF147650
A/Black-headed Gull/Netherlands/5/2013 H16N3 KX978693
A/Black-headed Gull/Netherlands/4/2013 H16N3 KX979151
A/Black-headed Gull/Netherlands/3/2013 H16N3 KX978186
A/Black-headed Gull/Netherlands/2/2013 H16N3 KX977836
A/Black-headed Gull/Netherlands/100/2008 H16N3 MF147121
A/Black-headed Gull/Netherlands/101/2008 H16N3 KX978252
A/Black-headed Gull/Netherlands/99/2008 H16N3 KX978955
A/Black-headed Gull/Netherlands/13/2011 H16N3 KX978398
A/Black-headed Gull/Netherlands/63/2008 H16N3 KX979453
A/Black-headed Gull/Netherlands/92/2008 H16N3 KX978323
A/Black-headed Gull/Netherlands/43/2008 H16N8 MF147250
A/Black-headed Gull/Netherlands/29/2008 H16N3 MF146193
A/Black-headed Gull/Netherlands/28/2008 H16N3 MF146238
A/Black-headed Gull/Netherlands/69/2008 H16N3 MF145725
A/Black-headed Gull/Netherlands/50/2008 H16N3 MF145888
A/Black-headed Gull/Netherlands/72/2008 H16N3 MF146541
A/Black-headed Gull/Netherlands/59/2008 H16N3 MF147239
A/Black-headed Gull/Netherlands/46/2008 H16N3 MF146816
A/Black-headed Gull/Netherlands/33/2008 H16N3 MF148040
A/Black-headed Gull/Netherlands/62/2008 H16N3 MF147547
A/Black-headed Gull/Netherlands/76/2008 H16N3 MF146737
A/Black-headed Gull/Netherlands/42/2008 H16N3 MF145764
A/Black-headed Gull/Netherlands/27/2008 H16N3 MF146654
A/Black-headed Gull/Netherlands/26/2008 H16N3 MF147314
A/Black-headed Gull/Sweden/9504/2005 H16N3 KR087608
A/Herring Gull/Norway/101623/2006 H16N3 FM179756
A/Black-headed Gull/Mongolia/1756/2006 H16N3 GQ907294
A/Black-headed Gull/Netherlands/14/2010 H16N3 KX977766
A/Black-headed Gull/Netherlands/9/2010 H16N3 KX978651
A/Black-headed Gull/Netherlands/12/2009 H16N3 MF147927
A/Black-headed Gull/Netherlands/15/2009 H16N3 MF146410
A/Black-headed Gull/Netherlands/19/2009 H16N3 MF146148
A/Black-headed Gull/Netherlands/26/2009 H16N3 KR087572
A/Black-headed Gull/Netherlands/14/2009 H16N3 KX978997
A/Black-headed Gull/Netherlands/23/2009 H16N3 MF146268
A/Black-headed Gull/Netherlands/22/2009 H16N3 KX978709
A/Great black-backed Gull/Netherlands/1/2008 H16N3 MF146805
A/Black-headed Gull/Republic of Georgia/4/2012 H16N3 CY185585
A/Black-headed Gull/Netherlands/37/2011 H16N3 MF146714
A/Black-headed Gull/Netherlands/5/2015 H16N3 KX977739
A/Black-headed Gull/Netherlands/2/2015 H16N3 KX978663
A/Black-headed Gull/Netherlands/3/2015 H16N3 KX978028
A/Black-headed Gull/Netherlands/4/2015 H16N3 KX978525
A/Environment/RhodeIsland/NWRC182872-06/2006 H16N3 CY122476
A/Environment/Alaska/NWRC184854-12/2006 H16N3 CY122507
A/Fulica atra/Volga/635/1986 H16N3 EU564109
A/Black-headed Gull/Turkmenistan/13/1976 H16N3 EU293864
A/Teal/Volga/671/1986 H16N3 EU148602
A/Slender-billed Gull/Astrakhan/28/1976 H16N3 EU293865
A/Black-headed Gull/Sweden/5/1999 H16N3 AY684891
A/Duck/Hokkaido/WZ82/2013 H16N3 LC339707
A/Little Tern/Sweden/55316/2006 H16N3 KR087616
A/wildbird/Sweden/1/2005 H16N3 KR087602
A/wildbird/Sweden/2/2005 H16N3 KR087603
A/Black-legged Kittiwake/Alaska/295/1975 H16N3 CY015160
A/Gull/MD/4985/79 H16-mixed KF612964
A/Herring Gull/DelawareBay/2617/1987 H16N3 CY136606
A/Laughing Gull/DelawareBay/2623/1987 H16N3 CY136614
A/Shorebird/NewJersey/840/1986 H16N3 CY014599
A/Herring Gull/NewJersey/780/1986 H16N3 CY136590
A/Laughing Gull/DelawareBay/2839/1987 H16N3 CY136630
A/Herring Gull/Delaware/712/1988 H16N3 CY136729
A/Gull/NJ/48/92 H16-mixed KF612961
A/Herring Gull/NJ/163/90 H16-mixed KF612962
A/Laughing Gull/DelawareBay/296/1998 H16N3 CY127445
A/Herring Gull/NewYork/AI00-532/2000 H16N3 CY144178
A/Waterfowl/GA/96623-7/01 H16N3 KF612963
A/Glaucous Gull/Alaska/44198-027/2006 H16N3 HM059998
A/Environment/Utah/NWRC184989-18/2006 H16N3 CY122509
A/Glaucous-winged Gull/SoutheasternAlaska/10JR01700R0/2010 H16N3 CY130509
A/Glaucous-winged Gull/SoutheasternAlaska/10JR01811R0/2010 H16N3 CY130517
A/Gull/SoutheasternAlaska/10JR01527R0/2010 H16N3 CY130485
A/Glaucous-winged Gull/SoutheasternAlaska/10JR01572R0/2010 H16N3 CY130493
A/Gull/Denmark/68110/2002 H16N3 GQ247872
A/Mallard/Quebec/02916-1/2009 H16 CY125606
A/Shorebird/Delaware/168/2006 H16N3 EU030976
A/Environment/NewHampshire/NWRC182016-06/2006 H16N3 CY122448
A/Environment/CO/492008/07 H16N3 KF612965
A/Common Gull/Norway/101617/2006 H16N3 FM179755
A/Herring Gull/Finland/13022/2005 H16 KX455114
A/European Herring Gull/Netherlands/5/2006 H16N3 MF147450
A/LittleTern/Sweden/8897/2005 H16N3 KR087604
A/Black-headed Gull/Netherlands/32/2009 H16N3 KX979111
A/Black-headed Gull/Netherlands/11/2010 H16N3 KX977650
A/European Herring Gull/Netherlands/1/2010 H16N3 KX979488
A/European Herring Gull/Netherlands/3/2015 H16N3 MF693968
A/Black-headed Gull/Netherlands/3/2010 H16N3 KX978612
A/Duck/Hokkaido/WZ82/2013 H16N3 AB937721
A/Lesser black-backed Gull/Netherlands/1/2015 H16N3 MF694110
A/Black-headed Gull/Netherlands/18/2014 H16N3 MF146608
A/European Herring Gull/Netherlands/2/2014 H16N3 MF147061
A/Black-headed Gull/Iceland/713/2010 H16N3 CY138145
A/Herring Gull/Newfoundland/GR032/2010 H16N3 KC845043
A/Brown-hooded gull/Chile/C8851/2016 H16N3 MH498904
A/Franklins gull/Chile/C10794/2016 H16N3 MH134685
A/Franklins gull/Chile/C10784/2016 H16N3 MH134702
A/Gull/Massachusetts/13WP00522/2013 H16N3 CY195833
A/Ruddy Turnstone/New Jersey/AI13-2872/2013 H16N3 MH502494
A/Gull/Massachusetts/12JR00662/2012 H16N3 CY195655
A/Laughing Gull/New Jersey/AI13-1937/2013 H16N3 MH501070
A/Gull/New Jersey/UGAI15-3414/2015 H16N3 MH501022
A/Gull/New Jersey/UGAI15-3459/2015 H16N3 MH501038
A/Environment/New Jersey/UGAI16-0887/2016 H16-mixed CY240896
A/Environment/New Jersey/UGAI16-1048/2016 H16N3 CY240948
A/Environment/New Jersey/UGAI16-0787/2016 H16N3 CY240828
A/Glaucous-winged Gull/Southcentral Alaska/11JR00366/2011 H16N3 CY196403
A/Glaucous-winged Gull/Southcentral Alaska/11JR01871/2011 H16N3 CY196563
A/Glaucous-winged Gull/Southcentral Alaska/11JR01859/2011 H16N3 CY196555
A/Glaucous-winged Gull/Southcentral Alaska/10JR01814/2010 H16N3 CY196009
A/Glaucous-winged Gull/Southcentral Alaska/11JR01852/2011 H16N3 CY196547
A/Glaucous-winged Gull/Southcentral Alaska/11JR02272/2011 H16N3 CY196603
A/Glaucous-winged Gull/Southcentral Alaska/11JR01722/2011 H16-mixed CY195614
A/Glaucous-winged Gull/Southcentral Alaska/11JR01732/2011 H16N3 CY196483
A/Environment/California/1242V/2012 H16N3 CY176997
A/Glaucous-winged Gull/Southcentral Alaska/12NH01538/2008 H16N3 CY196772
A/Glaucous-winged Gull/Southcentral Alaska/12NH01593/2008 H16N3 CY196788
A/Glaucous-winged Gull/Southcentral Alaska/12MB01812/2012 H16N3 CY195703
A/Glaucous-winged Gull/Southcentral Alaska/12NH01518/2008 H16N3 CY196764
A/Glaucous-winged Gull/Southcentral Alaska/12NH01679/2008 H16N3 CY196812
A/Glaucous-winged Gull/Southcentral Alaska/12NH01632/2012 H16N3 CY195793
A/Glaucous-winged Gull/Southcentral Alaska/12MB01573/2012 H16N3 CY195671
A/Glaucous-winged Gull/Southcentral Alaska/12MB01823/2012 H16N3 CY195711
A/Glaucous-winged Gull/Southcentral Alaska/12NH01285/2008 H16N3 CY196756
A/California Gull/California/1196P/2013 H16N3 CY177441
A/Glaucous-winged Gull/Southcentral Alaska/12NH01647/2008 H16N3 CY196804
A/Glaucous-winged Gull/Alaska/567/2013 H16N3 KY131088
A/Glaucous-winged Gull/Southcentral Alaska/13MB02558/2013 H16N3 CY213727
A/Glaucous-winged Gull/Southcentral Alaska/13MB02526/2013 H16N3 CY213711
A/Glaucous-winged Gull/Southcentral Alaska/13MB02527/2013 H16N3 CY213719
A/Glaucous-winged Gull/Southcentral Alaska/13MB02593/2013 H16N3 CY213759
A/Sandpiper/Southcentral Alaska/16MB01145/2016 H16-mixed CY213504
A/Glaucous-winged Gull/Southcentral Alaska/15MB01758/2015 H16N3 CY213679
A/Glaucous-winged Gull/Southcentral Alaska/15MB01735/2015 H16N3 CY213551
A/Glaucous-winged Gull/Southcentral Alaska/15MB01747/2015 H16N3 CY213671
A/Glaucous-winged Gull/Southcentral Alaska/15MB01680/2015 H16N3 CY213655
A/Northern pintail/Alaska/886/2014 H16N3 KT338585
A/Glaucous-winged Gull/Southcentral Alaska/16MB03160/2016 H16N3 CY239392
A/Glaucous-winged Gull/Southcentral Alaska/16MB02936/2016 H16N3 CY239320
A/Glaucous-winged Gull/Southcentral Alaska/16MB00033/2016 H16N3 CY239269
A/Glaucous-winged Gull/Southcentral Alaska/16MB02960/2016 H16N3 CY239344
A/Glaucous-winged Gull/Southcentral Alaska/16MB00031/2016 H16N3 CY239376
A/Glaucous-winged Gull/Southcentral Alaska/16MB03039/2016 H16N3 CY239360
A/Glaucous-winged Gull/Southcentral Alaska/16MB03027/2016 H16N3 CY239352
A/Glaucous-winged Gull/Southcentral Alaska/16MB03089/2016 H16N3 CY239384
A/Glaucous-winged Gull/Southcentral Alaska/14MB01770/2014 H16N3 CY206950
A/Glaucous-winged Gull/Southcentral Alaska/14MB01438/2014 H16N3 CY206926
A/Glaucous-winged Gull/Southcentral Alaska/14MB01417/2014 H16N3 CY206902
A/Glaucous-winged Gull/Southcentral Alaska/14MB01383/2014 H16N3 CY206886
A/Glaucous-winged Gull/Southcentral Alaska/14MB01615/2014 H16N3 CY206934
A/Glaucous-winged Gull/Southcentral Alaska/14MB01926/2014 H16N3 CY206990
A/Glaucous-winged Gull/Southcentral Alaska/14MB01959/2014 H16N3 CY206998
A/Glaucous-winged Gull/Southcentral Alaska/14MB01306/2014 H16N3 CY206870
A/Glaucous-winged Gull/Southcentral Alaska/14MB01884/2014 H16N3 CY206966
A/Glaucous-winged Gull/Southcentral Alaska/14MB01893/2014 H16N3 CY206982
A/Glaucous-winged Gull/Southcentral Alaska/14MB01444/2014 H16N3 CY239304
A/Glaucous-winged Gull/Southcentral Alaska/14MB01886/2014 H16N3 CY206974
A/Glaucous-winged Gull/Southcentral Alaska/14MB00623/2014 H16N3 CY206862
A/Glaucous-winged Gull/Southcentral Alaska/14MB01336/2014 H16N3 CY206677
A/Glaucous-winged Gull/Southcentral Alaska/14MB01318/2014 H16N3 CY206878
A/Glaucous-winged Gull/Southcentral Alaska/14MB02018/2014 H16N3 CY207006
0.2

Figure S5

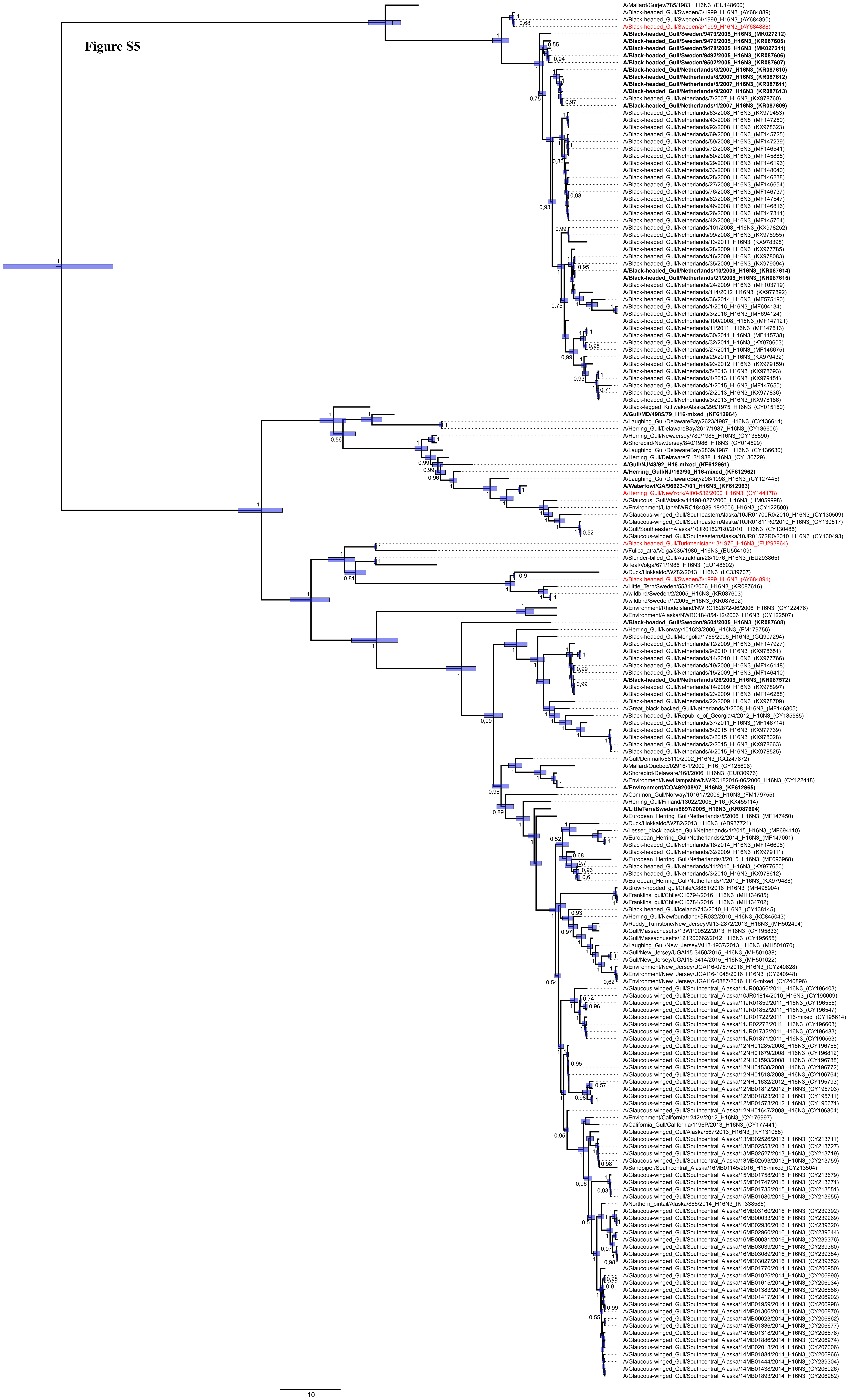
A/Mallard/Gurjev/785/1983_H16N3_(EU148600)
A/Black-headed_Gull/Sweden/3/1999_H16N3_(AY684889)
A/Black-headed_Gull/Sweden/4/1999_H16N3_(AY684890)
A/Black-headed_Gull/Sweden/2/1999_H16N3_(AY684888)
A/Black-headed_Gull/Sweden/9479/2005_H16N3_(MK027212)
A/Black-headed_Gull/Sweden/9476/2005_H16N3_(KR087605)
A/Black-headed_Gull/Sweden/9478/2005_H16N3_(MK027211)
A/Black-headed_Gull/Sweden/9492/2005_H16N3_(KR087606)
A/Black-headed_Gull/Sweden/9502/2005_H16N3_(KR087607)
A/Black-headed_Gull/Netherlands/3/2007_H16N3_(KR087610)
A/Black-headed_Gull/Netherlands/8/2007_H16N3_(KR087612)
A/Black-headed_Gull/Netherlands/5/2007_H16N3_(KR087611)
A/Black-headed_Gull/Netherlands/9/2007_H16N3_(KR087613)
A/Black-headed_Gull/Netherlands/7/2007_H16N3_(KX978760)
A/Black-headed_Gull/Netherlands/1/2007_H16N3_(KR087609)
A/Black-headed_Gull/Netherlands/63/2008_H16N3_(KX979453)
A/Black-headed_Gull/Netherlands/43/2008_H16N8_(MF147250)
A/Black-headed_Gull/Netherlands/92/2008_H16N3_(KX978323)
A/Black-headed_Gull/Netherlands/69/2008_H16N3_(MF145725)
A/Black-headed_Gull/Netherlands/59/2008_H16N3_(MF147239)
A/Black-headed_Gull/Netherlands/72/2008_H16N3_(MF146541)
A/Black-headed_Gull/Netherlands/50/2008_H16N3_(MF145888)
A/Black-headed_Gull/Netherlands/29/2008_H16N3_(MF146193)
A/Black-headed_Gull/Netherlands/33/2008_H16N3_(MF148040)
A/Black-headed_Gull/Netherlands/28/2008_H16N3_(MF146238)
A/Black-headed_Gull/Netherlands/27/2008_H16N3_(MF146654)
A/Black-headed_Gull/Netherlands/76/2008_H16N3_(MF146737)
A/Black-headed_Gull/Netherlands/62/2008_H16N3_(MF147547)
A/Black-headed_Gull/Netherlands/46/2008_H16N3_(MF146816)
A/Black-headed_Gull/Netherlands/26/2008_H16N3_(MF147314)
A/Black-headed_Gull/Netherlands/42/2008_H16N3_(MF145764)
A/Black-headed_Gull/Netherlands/101/2008_H16N3_(KX978252)
A/Black-headed_Gull/Netherlands/99/2008_H16N3_(KX978955)
A/Black-headed_Gull/Netherlands/13/2011_H16N3_(KX978398)
A/Black-headed_Gull/Netherlands/28/2009_H16N3_(KX977785)
A/Black-headed_Gull/Netherlands/16/2009_H16N3_(KX978083)
A/Black-headed_Gull/Netherlands/35/2009_H16N3_(KX979094)
A/Black-headed_Gull/Netherlands/10/2009_H16N3_(KR087614)
A/Black-headed_Gull/Netherlands/21/2009_H16N3_(KR087615)
A/Black-headed_Gull/Netherlands/24/2009_H16N3_(MF103719)
A/Black-headed_Gull/Netherlands/114/2012_H16N3_(KX977892)
A/Black-headed_Gull/Netherlands/36/2014_H16N3_(MF575190)
A/Black-headed_Gull/Netherlands/1/2016_H16N3_(MF694134)
A/Black-headed_Gull/Netherlands/3/2016_H16N3_(MF694124)
A/Black-headed_Gull/Netherlands/100/2008_H16N3_(MF147121)
A/Black-headed_Gull/Netherlands/11/2011_H16N3_(MF147513)
A/Black-headed_Gull/Netherlands/30/2011_H16N3_(MF145738)
A/Black-headed_Gull/Netherlands/32/2011_H16N3_(KX979603)
A/Black-headed_Gull/Netherlands/27/2011_H16N3_(MF146675)
A/Black-headed_Gull/Netherlands/29/2011_H16N3_(KX979432)
A/Black-headed_Gull/Netherlands/93/2012_H16N3_(KX979159)
A/Black-headed_Gull/Netherlands/5/2013_H16N3_(KX978693)
A/Black-headed_Gull/Netherlands/4/2013_H16N3_(KX979151)
A/Black-headed_Gull/Netherlands/1/2015_H16N3_(MF147650)
A/Black-headed_Gull/Netherlands/2/2013_H16N3_(KX977836)
A/Black-headed_Gull/Netherlands/3/2013_H16N3_(KX978186)
A/Black-legged_Kittiwake/Alaska/295/1975_H16N3_(CY015160)
A/Gull/MD/4985/79_H16-mixed_(KF612964)
A/Laughing_Gull/DelawareBay/2623/1987_H16N3_(CY136614)
A/Herring_Gull/DelawareBay/2617/1987_H16N3_(CY136606)
A/Herring_Gull/NewJersey/780/1986_H16N3_(CY136590)
A/Shorebird/NewJersey/840/1986_H16N3_(CY014599)
A/Laughing_Gull/DelawareBay/2839/1987_H16N3_(CY136630)
A/Herring_Gull/Delaware/712/1988_H16N3_(CY136729)
A/Gull/NJ/48/92_H16-mixed_(KF612961)
A/Herring_Gull/NJ/163/90_H16-mixed_(KF612962)
A/Laughing_Gull/DelawareBay/296/1998_H16N3_(CY127445)
A/Waterfowl/GA/96623-7/01_H16N3_(KF612963)
A/Herring_Gull/NewYork/AI00-532/2000_H16N3_(CY144178)
A/Glaucous_Gull/Alaska/44198-027/2006_H16N3_(HM059998)
A/Environment/Utah/NWRC184989-18/2006_H16N3_(CY122509)
A/Glaucous-winged_Gull/SoutheasternAlaska/10JR01700R0/2010_H16N3_(CY130509)
A/Glaucous-winged_Gull/SoutheasternAlaska/10JR01811R0/2010_H16N3_(CY130517)
A/Gull/SoutheasternAlaska/10JR01527R0/2010_H16N3_(CY130485)
A/Glaucous-winged_Gull/SoutheasternAlaska/10JR01572R0/2010_H16N3_(CY130493)
A/Black-headed_Gull/Turkmenistan/13/1976_H16N3_(EU293864)
A/Fulica_atra/Volga/635/1986_H16N3_(EU564109)
A/Slender-billed_Gull/Astrakhan/28/1976_H16N3_(EU293865)
A/Teal/Volga/671/1986_H16N3_(EU148602)
A/Duck/Hokkaido/WZ82/2013_H16N3_(LC339707)
A/Black-headed_Gull/Sweden/5/1999_H16N3_(AY684891)
A/Little_Tern/Sweden/55316/2006_H16N3_(KR087616)
A/wildbird/Sweden/2/2005_H16N3_(KR087603)
A/wildbird/Sweden/1/2005_H16N3_(KR087602)
A/Environment/RhodeIsland/NWRC182872-06/2006_H16N3_(CY122476)
A/Environment/Alaska/NWRC184854-12/2006_H16N3_(CY122507)
A/Black-headed_Gull/Sweden/9504/2005_H16N3_(KR087608)
A/Herring_Gull/Norway/101623/2006_H16N3_(FM179756)
A/Black-headed_Gull/Mongolia/1756/2006_H16N3_(GQ907294)
A/Black-headed_Gull/Netherlands/12/2009_H16N3_(MF147927)
A/Black-headed_Gull/Netherlands/9/2010_H16N3_(KX978651)
A/Black-headed_Gull/Netherlands/14/2010_H16N3_(KX977766)
A/Black-headed_Gull/Netherlands/19/2009_H16N3_(MF146148)
A/Black-headed_Gull/Netherlands/15/2009_H16N3_(MF146410)
A/Black-headed_Gull/Netherlands/26/2009_H16N3_(KR087572)
A/Black-headed_Gull/Netherlands/14/2009_H16N3_(KX978997)
A/Black-headed_Gull/Netherlands/23/2009_H16N3_(MF146268)
A/Black-headed_Gull/Netherlands/22/2009_H16N3_(KX978709)
A/Great_black-backed_Gull/Netherlands/1/2008_H16N3_(MF146805)
A/Black-headed_Gull/Republic_of_Georgia/4/2012_H16N3_(CY185585)
A/Black-headed_Gull/Netherlands/37/2011_H16N3_(MF146714)
A/Black-headed_Gull/Netherlands/5/2015_H16N3_(KX977739)
A/Black-headed_Gull/Netherlands/3/2015_H16N3_(KX978028)
A/Black-headed_Gull/Netherlands/2/2015_H16N3_(KX978663)
A/Black-headed_Gull/Netherlands/4/2015_H16N3_(KX978525)
A/Gull/Denmark/68110/2002_H16N3_(GQ247872)
A/Mallard/Quebec/02916-1/2009_H16_(CY125606)
A/Shorebird/Delaware/168/2006_H16N3_(EU030976)
A/Environment/NewHampshire/NWRC182016-06/2006_H16N3_(CY122448)
A/Environment/CO/492008/07_H16N3_(KF612965)
A/Common_Gull/Norway/101617/2006_H16N3_(FM179755)
A/Herring_Gull/Finland/13022/2005_H16_(KX455114)
A/LittleTern/Sweden/8897/2005_H16N3_(KR087604)
A/European_Herring_Gull/Netherlands/5/2006_H16N3_(MF147450)
A/Duck/Hokkaido/WZ82/2013_H16N3_(AB937721)
A/Lesser_black-backed_Gull/Netherlands/1/2015_H16N3_(MF694110)
A/European_Herring_Gull/Netherlands/2/2014_H16N3_(MF147061)
A/Black-headed_Gull/Netherlands/18/2014_H16N3_(MF146608)
A/Black-headed_Gull/Netherlands/32/2009_H16N3_(KX979111)
A/European_Herring_Gull/Netherlands/3/2015_H16N3_(MF693968)
A/Black-headed_Gull/Netherlands/11/2010_H16N3_(KX977650)
A/Black-headed_Gull/Netherlands/3/2010_H16N3_(KX978612)
A/European_Herring_Gull/Netherlands/1/2010_H16N3_(KX979488)
A/Brown-hooded_gull/Chile/C8851/2016_H16N3_(MH498904)
A/Franklins_gull/Chile/C10794/2016_H16N3_(MH134685)
A/Franklins_gull/Chile/C10784/2016_H16N3_(MH134702)
A/Black-headed_Gull/Iceland/713/2010_H16N3_(CY138145)
A/Herring_Gull/Newfoundland/GR032/2010_H16N3_(KC845043)
A/Ruddy_Turnstone/New_Jersey/AI13-2872/2013_H16N3_(MH502494)
A/Gull/Massachusetts/13WP00522/2013_H16N3_(CY195833)
A/Gull/Massachusetts/12JR00662/2012_H16N3_(CY195655)
A/Laughing_Gull/New_Jersey/AI13-1937/2013_H16N3_(MH501070)
A/Gull/New_Jersey/UGAI15-3459/2015_H16N3_(MH501038)
A/Gull/New_Jersey/UGAI15-3414/2015_H16N3_(MH501022)
A/Environment/New_Jersey/UGAI16-0787/2016_H16N3_(CY240828)
A/Environment/New_Jersey/UGAI16-1048/2016_H16N3_(CY240948)
A/Environment/New_Jersey/UGAI16-0887/2016_H16-mixed_(CY240896)
A/Glaucous-winged_Gull/Southcentral_Alaska/11JR00366/2011_H16N3_(CY196403)
A/Glaucous-winged_Gull/Southcentral_Alaska/10JR01814/2010_H16N3_(CY196009)
A/Glaucous-winged_Gull/Southcentral_Alaska/11JR01859/2011_H16N3_(CY196555)
A/Glaucous-winged_Gull/Southcentral_Alaska/11JR01852/2011_H16N3_(CY196547)
A/Glaucous-winged_Gull/Southcentral_Alaska/11JR01722/2011_H16-mixed_(CY195614)
A/Glaucous-winged_Gull/Southcentral_Alaska/11JR02272/2011_H16N3_(CY196603)
A/Glaucous-winged_Gull/Southcentral_Alaska/11JR01732/2011_H16N3_(CY196483)
A/Glaucous-winged_Gull/Southcentral_Alaska/11JR01871/2011_H16N3_(CY196563)
A/Glaucous-winged_Gull/Southcentral_Alaska/12NH01285/2008_H16N3_(CY196756)
A/Glaucous-winged_Gull/Southcentral_Alaska/12NH01679/2008_H16N3_(CY196812)
A/Glaucous-winged_Gull/Southcentral_Alaska/12NH01593/2008_H16N3_(CY196788)
A/Glaucous-winged_Gull/Southcentral_Alaska/12NH01538/2008_H16N3_(CY196772)
A/Glaucous-winged_Gull/Southcentral_Alaska/12NH01518/2008_H16N3_(CY196764)
A/Glaucous-winged_Gull/Southcentral_Alaska/12NH01632/2012_H16N3_(CY195793)
A/Glaucous-winged_Gull/Southcentral_Alaska/12MB01812/2012_H16N3_(CY195703)
A/Glaucous-winged_Gull/Southcentral_Alaska/12MB01823/2012_H16N3_(CY195711)
A/Glaucous-winged_Gull/Southcentral_Alaska/12MB01573/2012_H16N3_(CY195671)
A/Glaucous-winged_Gull/Southcentral_Alaska/12NH01647/2008_H16N3_(CY196804)
A/Environment/California/1242V/2012_H16N3_(CY176997)
A/California_Gull/California/1196P/2013_H16N3_(CY177441)
A/Glaucous-winged_Gull/Alaska/567/2013_H16N3_(KY131088)
A/Glaucous-winged_Gull/Southcentral_Alaska/13MB02526/2013_H16N3_(CY213711)
A/Glaucous-winged_Gull/Southcentral_Alaska/13MB02558/2013_H16N3_(CY213727)
A/Glaucous-winged_Gull/Southcentral_Alaska/13MB02527/2013_H16N3_(CY213719)
A/Glaucous-winged_Gull/Southcentral_Alaska/13MB02593/2013_H16N3_(CY213759)
A/Sandpiper/Southcentral_Alaska/16MB01445/2016_H16-mixed_(CY213504)
A/Glaucous-winged_Gull/Southcentral_Alaska/15MB01758/2015_H16N3_(CY213679)
A/Glaucous-winged_Gull/Southcentral_Alaska/15MB01747/2015_H16N3_(CY213671)
A/Glaucous-winged_Gull/Southcentral_Alaska/15MB01735/2015_H16N3_(CY213551)
A/Glaucous-winged_Gull/Southcentral_Alaska/15MB01680/2015_H16N3_(CY213655)
A/Northern_pintail/Alaska/886/2014_H16N3_(KT338585)
A/Glaucous-winged_Gull/Southcentral_Alaska/16MB03160/2016_H16N3_(CY239392)
A/Glaucous-winged_Gull/Southcentral_Alaska/16MB00033/2016_H16N3_(CY239269)
A/Glaucous-winged_Gull/Southcentral_Alaska/16MB02936/2016_H16N3_(CY239320)
A/Glaucous-winged_Gull/Southcentral_Alaska/16MB02960/2016_H16N3_(CY239344)
A/Glaucous-winged_Gull/Southcentral_Alaska/16MB00031/2016_H16N3_(CY239376)
A/Glaucous-winged_Gull/Southcentral_Alaska/16MB03039/2016_H16N3_(CY239360)
A/Glaucous-winged_Gull/Southcentral_Alaska/16MB03089/2016_H16N3_(CY239384)
A/Glaucous-winged_Gull/Southcentral_Alaska/16MB03027/2016_H16N3_(CY239352)
A/Glaucous-winged_Gull/Southcentral_Alaska/14MB01770/2014_H16N3_(CY206950)
A/Glaucous-winged_Gull/Southcentral_Alaska/14MB01926/2014_H16N3_(CY206990)
A/Glaucous-winged_Gull/Southcentral_Alaska/14MB01615/2014_H16N3_(CY206934)
A/Glaucous-winged_Gull/Southcentral_Alaska/14MB01383/2014_H16N3_(CY206886)
A/Glaucous-winged_Gull/Southcentral_Alaska/14MB01417/2014_H16N3_(CY206902)
A/Glaucous-winged_Gull/Southcentral_Alaska/14MB01959/2014_H16N3_(CY206998)
A/Glaucous-winged_Gull/Southcentral_Alaska/14MB01306/2014_H16N3_(CY206870)
A/Glaucous-winged_Gull/Southcentral_Alaska/14MB00623/2014_H16N3_(CY206862)
A/Glaucous-winged_Gull/Southcentral_Alaska/14MB01336/2014_H16N3_(CY206677)
A/Glaucous-winged_Gull/Southcentral_Alaska/14MB01318/2014_H16N3_(CY206878)
A/Glaucous-winged_Gull/Southcentral_Alaska/14MB01886/2014_H16N3_(CY206974)
A/Glaucous-winged_Gull/Southcentral_Alaska/14MB02018/2014_H16N3_(CY207006)
A/Glaucous-winged_Gull/Southcentral_Alaska/14MB01884/2014_H16N3_(CY206966)
A/Glaucous-winged_Gull/Southcentral_Alaska/14MB01444/2014_H16N3_(CY239304)
A/Glaucous-winged_Gull/Southcentral_Alaska/14MB01438/2014_H16N3_(CY206926)
A/Glaucous-winged_Gull/Southcentral_Alaska/14MB01893/2014_H16N3_(CY206982)
10

Figure S6

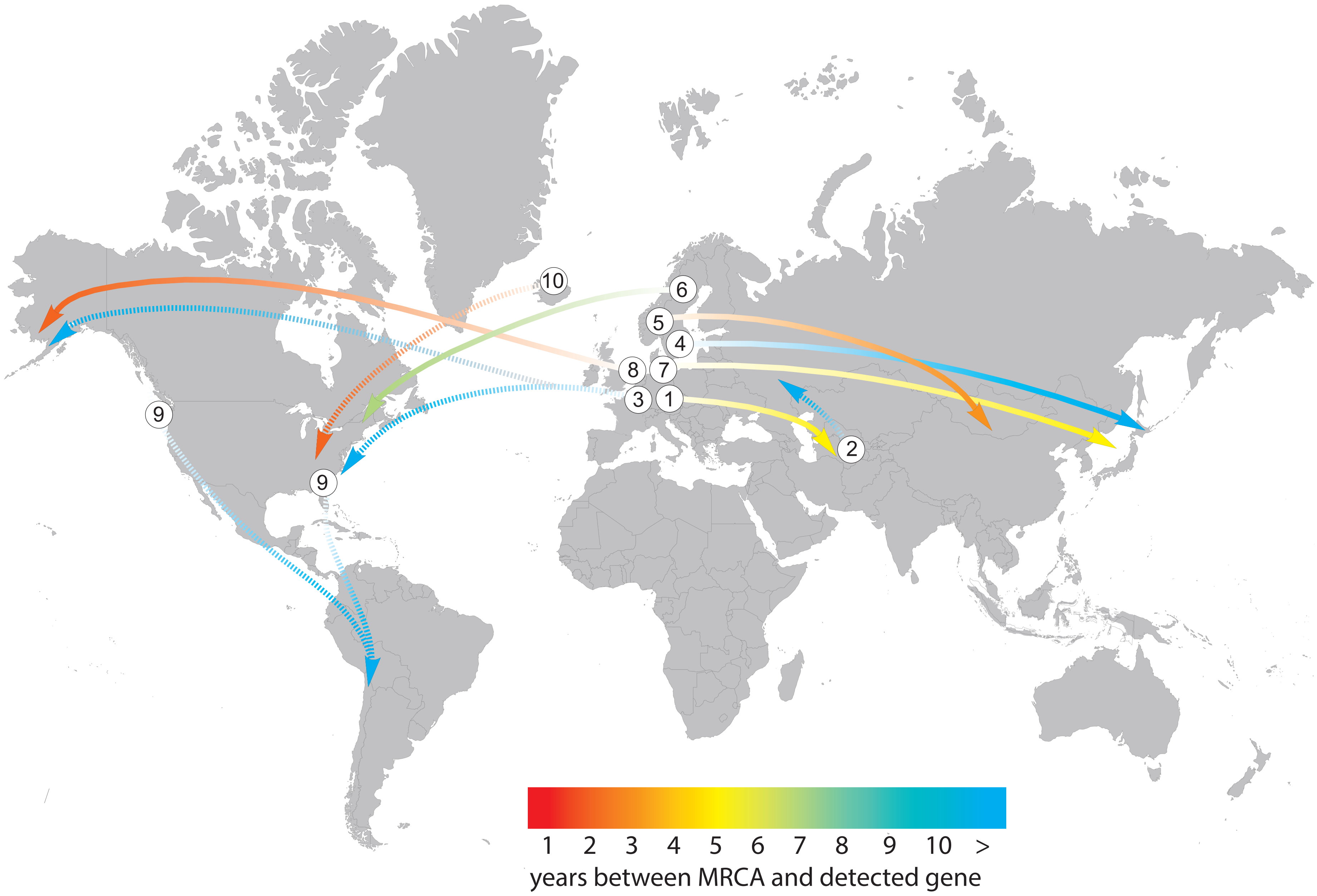

10
6
5
4
8
7
3
1
2
9
9
1 2 3 4 5 6 7 8 9 10 >
years between MRCA and detected gene

Tabel S1

Host		Region	No. Subtype (%)																Total
Common name	Latin name		H1	H2	H3	H4	H5	H6	H7	H8	H9	H10	H11	H12	H13	H14	H15	H16	
Armenian gull	*Larus armenicus*	Eurasia	-	-	-	-	-	-	-	-	-	-	-	-	2 (100)	-	-	-	2 (100)
		America	-	-	-	-	-	-	-	-	-	-	-	-	-	-	-	-	-
Black-headed gull	*Chroicocephalus ridibundus*	Eurasia	1 (0)	3 (1)	1 (0)	1 (0)	8 (2)	1 (0)	-	-	5 (1)	1 (0)	3 (1)	-	334 (70)	-	-	119 (25)	477 (100)
		America	-	-	-	-	-	-	-	-	-	-	-	-	-	-	-	-	-
Black-tailed gull	*Larus crassirostris*	Eurasia	-	-	-	-	-	-	-	-	-	-	-	-	4 (100)	-	-	-	4 (100)
		America	-	-	-	-	-	-	-	-	-	-	-	-	-	-	-	-	-
Brown-Headed gull	*Chroicocephalus brunnicephalus*	Eurasia	-	-	-	-	11 (100)	-	-	-	-	-	-	-	-	-	-	-	11 (100)
		America	-	-	-	-	-	-	-	-	-	-	-	-	-	-	-	-	-
Brown-hooded gull	*Chroicocephalus maculipennis*	Eurasia	-	-	-	-	-	-	-	-	-	-	-	-	-	-	-	-	-
		America	-	-	-	-	-	-	-	-	1 (20)	-	-	-	3 (60)	-	-	1 (20)	5 (100)
California gull	*Larus californicus*	Eurasia	-	-	-	-	-	-	-	-	-	-	-	-	-	-	-	-	-
		America	-	-	-	-	-	-	-	-	-	1 (50)	-	-	-	-	-	1 (50)	2 (100)
Dolphin gull	*Leucophaeus scoresbii*	Eurasia	-	-	-	-	-	-	-	-	-	-	-	-	-	-	-	-	-
		America	-	-	-	-	-	-	-	-	-	-	-	-	-	-	-	1 (100)	1 (100)
Franklin's gull	*Leucophaeus pipixcan*	Eurasia	-	-	-	-	-	-	-	-	-	-	-	-	-	-	-	-	-
		America	-	-	-	-	-	1 (7)	-	-	-	-	-	-	11 (79)	-	-	2 (14)	14 (100)
Glaucous gull	*Larus hyperboreus*	Eurasia	-	2 (50)	-	-	-	-	-	-	-	2 (50)	-	-	-	-	-	-	4 (100)
		America	-	-	1 (13)	-	1 (13)	1 (13)	-	-	-	-	-	-	4 (50)	-	-	1 (13)	8 (100)
Glaucous-winged gull	*Larus glaucescens*	Eurasia	-	-	-	-	-	-	-	-	-	-	-	-	-	-	-	-	-
		America	1 (1)	-	4 (3)	3 (2)	4 (3)	-	-	-	-	-	2 (1)	-	41 (26)	-	-	101 (65)	156 (100)
Great black-backed gull	*Larus marinus*	Eurasia	-	2 (33)	-	1 (17)	1 (17)	-	-	-	-	-	-	-	1 (17)	-	-	1 (17)	6 (100)
		America	1 (20)	-	-	-	-	-	-	-	1 (20)	-	-	-	2 (40)	-	-	1 (20)	5 (100)
Great black-headed gull (Pallas's gull)	*Ichthyaetus ichthyaetus*	Eurasia	-	-	-	-	12 (55)	-	-	-	-	-	-	-	10 (45)	-	-	-	22 (100)
		America	-	-	-	-	-	-	-	-	-	-	-	-	-	-	-	-	-
Herring gull	*Larus argentatus*	America	1 (2)	7 (13)	1 (2)	1 (2)	3 (6)	3 (6)	-	-	-	3 (6)	4 (8)	-	22 (42)	-	-	8 (15)	53 (100)
		Eurasia	-	5 (12)	1 (2)	1 (2)	6 (14)	1 (2)	-	-	-	1 (2)	2 (5)	-	14 (33)	1 (2)	-	11 (26)	43 (100)
Iceland gull	*Larus glaucoides*	Eurasia	-	1 (33)	-	-	-	-	-	-	-	2 (67)	-	-	-	-	-	-	3 (100)
		America	-	-	-	-	-	-	-	-	-	-	-	-	-	-	-	-	-
Kelp gull	*Larus dominicanus*	Eurasia	-	-	-	-	-	-	-	-	-	-	-	-	-	-	-	-	-
		America	-	-	1 (4)	-	2 (8)	3 (12)	-	-	-	-	-	-	13 (52)	-	-	6 (24)	25 (100)
Laughing gull	*Leucophaeus atricilla*	Eurasia	-	-	-	-	-	-	-	-	-	-	-	-	-	-	-	-	-
		America	2 (2)	7 (7)	8 (8)	1 (1)	3 (3)	16 (16)	9 (9)	-	7 (7)	4 (4)	7 (7)	2 (2)	27 (27)	-	-	8 (8)	101 (100)
Little gull	*Hydrocoloeus minutus*	Eurasia	-	-	-	-	-	-	-	-	-	-	-	-	1 (100)	-	-	-	1 (100)
		America	-	-	-	-	-	-	-	-	-	-	-	-	-	-	-	-	-
Mediterranean gull	*Ichthyaetus melanocephalus*	Eurasia	-	-	-	-	-	-	-	-	3 (75)	-	-	-	1 (25)	-	-	-	4 (100)
		America	-	-	-	-	-	-	-	-	-	-	-	-	-	-	-	-	-
Mew Gull	*Larus canus*	Eurasia	-	-	-	-	1 (10)	1 (10)	-	-	-	-	-	-	4 (40)	-	-	4 (40)	10 (100)
		America	-	-	-	-	-	-	-	-	-	-	-	-	-	-	-	1 (100)	1 (100)
Ring-billed gull	*Larus delawarensis*	Eurasia	-	-	-	-	-	-	-	-	-	-	-	-	-	-	-	-	-
		America	5 (7)	-	-	-	-	3 (4)	-	-	-	-	2 (3)	-	55 (82)	-	-	2 (3)	67 (100)
Sabine'S gull	*Xema sabini*	Eurasia	-	-	-	-	-	-	-	-	-	-	-	-	-	-	-	-	-
		America	-	-	-	-	1 (100)	-	-	-	-	-	-	-	-	-	-	-	1 (100)
Slaty-backed gull	*Larus schistisagus*	Eurasia	-	-	-	2 (50)	2 (50)	-	-	-	-	-	-	-	-	-	-	-	4 (100)
		America	-	-	-	-	-	-	-	-	-	-	-	1 (100)	-	-	-	-	1 (100)
Slender-Billed gull	*Chroicocephalus genei*	Eurasia	-	-	-	-	-	-	-	-	-	-	-	-	1 (50)	-	-	1 (50)	2 (100)
		America	-	-	-	-	-	-	-	-	-	-	-	-	-	-	-	-	-
Yellow-legged gull	*Larus michahellis*	Eurasia	-	-	-	-	-	-	-	-	-	-	-	-	13 (100)	-	-	-	13 (100)
		America	-	-	-	-	-	-	-	-	-	-	-	-	-	-	-	-	-
Unknown gull species	Unknown gull species	Eurasia	-	-	2 (7)	2 (7)	3 (10)	2 (7)	3 (10)	-	-	-	2 (7)	-	13 (43)	-	-	3 (10)	30 (100)
		America	5 (6)	5 (6)	1 (1)	13 (16)	3 (4)	3 (4)	2 (3)	-	1 (1)	6 (8)	4 (5)	-	30 (38)	-	-	7 (9)	80 (100)
Total			16 (1)	32 (3)	20 (2)	25 (2)	61 (5)	35 (3)	14 (1)	-	18 (2)	20 (2)	26 (2)	-	606 (52)	1 (0)	-	279 (24)	1156 (100)

Table S2

Virus	Subtype	Accession number
**A/Gull/MD/704/77**	**H13N6**	**KF612959**
**A/Gull/MD/1815/78**	**H13N6**	**KF612933**
**A/Gull/MD/3027/78**	**H13N9**	**KF612945**
A/Great Black-headed Gull/Astrakhan/1420/1979	H13N2	EU293858
A/Great Black-headed Gull/Astrakhan/1421/1979	H13N2	EU293859
A/Gull/Astrakhan/1314/1979	H13N2	EU835898
**A/Gull/MD/4909/79**	**H13N6**	**KF612947**
**A/Gull/MD/4985/79**	**H13-mixed**	**KF612942**
**A/Gull/MA/18/80**	**H13N6**	**KF612943**
**A/Gull/MD/5049/80**	**H13N6**	**KF612941**
A/Gull/Minnesota/945/1980	H13N6	CY014720
**A/Gull/MN/1352/81**	**H13N6**	**KF612944**
A/Great Black-headed Gull/Astrakhan/591/1982	H13N2	EU293860
A/Black-headed Gull/Astrakhan/65/1983	H13N6	EU580577
A/Great Black-headed Gull/Gurjev/76/1983	H13N2	EU293861
A/Larus ichthyaetus/Astrakhan/75/1983	H13N2	EU564107
A/Black-headed Gull/Astrakhan/227/1984	H13N6	M26089
A/Gull/Astrakhan/226/1984	H13N6	EU835895
A/Pilot Whale/Maine/328HN/1984	H13N2	M26091
**A/Gull/ME/16/85**	**H13N2**	**KF612946**
A/Herring Gull/Astrakhan/458/1985	H13N6	EU293862
A/Herring Gull/Astrakhan/479/1985	H13N6	EU293863
A/Gull/Astrakhan/176/1986	H13N2	EU835899
**A/Herring Gull/DE/471/86**	**H13N7**	**KF612934**
A/Herring Gull/DE/475/1986	H13N2	CY005914
A/Herring Gull/NJ/782/1986	H13N2	CY005932
**A/Laughing Gull/DE/1370/86**	**H13N2**	**KF612923**
**A/Crab egg/DE/2618/87**	**H13N2**	**KF612922**
**A/Herring Gull/DE/2591/87**	**H13N2**	**KF612930**
**A/Laughing Gull/DE/2424/87**	**H13N2**	**KF612939**
A/Laughing Gull/DelawareBay/2838/1987	H13N2	CY101422
**A/Ruddy Turnstone/DE/2584/87**	**H13N2**	**KF612927**
**A/Sandpiper/DE/2516/87**	**H13N2**	**KF612951**
**A/Crab egg/DE/2347/88**	**H13N6**	**KF612928**
**A/Herring Gull/DE/2337/88**	**H13N2**	**KF612924**
A/Herring Gull/Delaware/660/1988	H13N6	CY014603
**A/Knot/DE/530/88**	**H13N6**	**KF612925**
A/Larus ichthyaetus/Astrakhan/10/1988	H13N6	EU564106
A/Larus ichthyaetus/Astrakhan/44/1988	H13N6	EU564115
**A/Laughing Gull/DE/554/88**	**H13N3**	**KF612926**
A/RuddyTurnstone/DelawareBay/520/1988	H13N9	CY126288
**A/Red Knot/NJ/321/89**	**H13N4**	**KF612940**
A/Gull/Astrakhan/998/1990	H13N6	EU835896
**A/Herring Gull/DE/13/90**	**H13N2**	**KF612938**
**A/Herring Gull/NJ/163/90**	**H13-mixed**	**KF612929**
**A/Turkey/MN/1012/91**	**H13N2**	**KF612932**
**A/Gull/ND/44036/92**	**H13N6**	**KF612960**
**A/Gull/NJ/34/92**	**H13N6**	**KF612936**
**A/Gull/NJ/48/92**	**H13-mixed**	**KF612935**
**A/Laughing Gull/DE/246/93**	**H13N6**	**KF612931**
**A/Laughing Gull/DE/254/93**	**H13N1**	**KF612937**
**A/Ruddy Turnstone/DE/179/94**	**H13N3**	**KF612955**
**A/Shorebird/DE/224/97**	**H13N6**	**KF612952**
A/Duck/Siberia/272/1998	H13N6	AB284988
A/Gull/Astrakhan/1818/1998	H13N6	EU835900
A/Gull/Astrakhan/1846/1998	H13N6	EU580576
A/Black-headed Gull/Sweden/1/1999	H13N6	AY684887
A/Black-headed Gull/Netherlands/1/2000	H13N8	MF146968
A/Ring-billed Gull/Georgia/AI00-2658/2000	H13N6	CY144202

Virus	Subtype	Accession number
**A/Shorebird/DE/188/2000**	**H13N6**	**KF612948**
A/Gull/Astrakhan/3483/2002	H13N6	EU835897
A/Larus minutus/Astrakhan/3357/2002	H13N2	EU564108
**A/Black-headed Gull/Sweden/1/2003**	**H13N8**	**KR087599**
**A/Black-headed Gull/Sweden/2/2003**	**H13N8**	**KR087600**
A/Great black-backed Gull/**Sweden/1/2003**	**H13N6**	**KR087577**
A/Great Black-headed Gull/Atyrau/743/2004	H13N6	GU982281
A/Great Black-headed Gull/Atyrau/744/2004	H13N6	GU982282
A/Great Black-headed Gull/Atyrau/767/2004	H13N6	GU982283
A/Great Black-headed Gull/Atyrau/773/2004	H13N6	GU982284
A/Shorebird/DE/68/2004	H13N9	CY005931
A/Black-headed Gull/Sweden/1/2005	H13N8	CY077000
**A/Black-headed Gull/Sweden/10/2005**	**H13N6**	**KR087578**
A/Herring Gull/Finland/9875/2005	H13	KX455108
A/Black-headed Gull/Mongolia/1766/2006	H13N6	GQ907302
**A/Black-headed Gull/Sweden/1/2006**	**H13N8**	**KR087597**
A/Duck/Hokkaido/W189/2006	H13N6	LC339627
A/Environment/Alabama/NWRC183838-18/2006	H13N2	CY122492
A/Environment/Florida/NWRC183796-24/2006	H13N2	CY122491
A/Environment/Georgia/NWRC183417-30/2006	H13N2	CY122488
A/Environment/Georgia/NWRC184017-60/2006	H13N2	CY122502
A/Environment/NewYork/NWRC182181-12/2006	H13N2	CY122459
A/Environment/Ohio/NWRC182318-30/2006	H13N2	CY122462
A/Glaucous Gull/Alaska/44199-006/2006	H13N9	HM059994
A/Glaucous Gull/Alaska/44199-097/2006	H13N3	HM059995
A/Glaucous Gull/Alaska/44199-104/2006	H13N9	HM059996
A/Herring Gull/Massachusetts/A00080255/2006	H13N2	CY239408
A/Herring Gull/Massachusetts/A00080257/2006	H13N2	CY239280
A/Herring Gull/Norway/102336/2006	H13N6	FM179758
A/Kelp Gull/Argentina/LDC4/2006	H13N9	EU523136
A/Lesser snow Goose/Alaska/44199-115/2006	H13N9	HM059997
A/Shorebird/Delaware/221/2006	H13N9	CY043888
A/Shorebird/Delaware/224/2006	H13N9	CY043896
A/SilverGull/Tasmania/62/2006	H13N6	CY094903
A/American White Pelican/Minnesota/AI-07-1819/2007	H13N9	CY054300
**A/Black-headed Gull/Netherlands/10/2007**	**H13N6**	**KR087582**
**A/Black-headed Gull/Netherlands/2/2007**	**H13N6**	**KR087579**
**A/Black-headed Gull/Netherlands/4/2007**	**H13N6**	**KR087580**
**A/Black-headed Gull/Netherlands/6/2007**	**H13N3**	**KR087581**
**A/Herring Gull/CT/1783-10/07**	**H13N3**	**KF612954**
A/Herring Gull/Finland/9330/2007	H13	KX455109
A/Herring Gull/Finland/9611/2007	H13	KX455110
A/Mongolian Gull/Mongolia/401/2007	H13N6	GQ907310
A/Mongolian Gull/Mongolia/405/2007	H13N6	GQ907318
A/Shorebird/DelawareBay/424/2007	H13N9	CY127799
A/American White Pelican/Minnesota/Sg-0611/2008	H13N9	CY054302
A/Black-headed Gull/Netherlands/10/2008	H13N8	MF682786
A/Black-headed Gull/Netherlands/102/2008	H13N8	KX978024
A/Black-headed Gull/Netherlands/11/2008	H13N8	MF147313
A/Black-headed Gull/Netherlands/12/2008	H13N8	MF146078
A/Black-headed Gull/Netherlands/13/2008	H13N8	MF148122
A/Black-headed Gull/Netherlands/14/2008	H13N8	MF145859
A/Black-headed Gull/Netherlands/15/2008	H13N8	MF146408
A/Black-headed Gull/Netherlands/16/2008	H13N8	MF146171
A/Black-headed Gull/Netherlands/17/2008	H13N8	MF146229
A/Black-headed Gull/Netherlands/18/2008	H13N8	MF147099
A/Black-headed Gull/Netherlands/19/2008	H13N8	MF146117
A/Black-headed Gull/Netherlands/2/2008	H13N8	MF146364
A/Black-headed Gull/Netherlands/20/2008	H13N8	KX977714

Virus	Subtype	Accession number
A/Black-headed Gull/Netherlands/21/2008	H13N8	KX978810
A/Black-headed Gull/Netherlands/22/2008	H13N8	MF145892
A/Black-headed Gull/Netherlands/23/2008	H13N8	KX978852
A/Black-headed Gull/Netherlands/24/2008	H13N8	MF147255
A/Black-headed Gull/Netherlands/25/2008	H13N8	KX979286
A/Black-headed Gull/Netherlands/3/2008	H13N8	MF146870
A/Black-headed Gull/Netherlands/30/2008	H13N8	MF146174
A/Black-headed Gull/Netherlands/35/2008	H13N8	MF682817
A/Black-headed Gull/Netherlands/37/2008	H13N8	MF146360
A/Black-headed Gull/Netherlands/38/2008	H13N8	MF146262
A/Black-headed Gull/Netherlands/39/2008	H13N8	MF148072
**A/Black-headed Gull/Netherlands/4/2008**	**H13N8**	**KR087601**
A/Black-headed Gull/Netherlands/44/2008	H13N8	MF146202
A/Black-headed Gull/Netherlands/48/2008	H13N8	MF682688
A/Black-headed Gull/Netherlands/49/2008	H13N8	MF147079
A/Black-headed Gull/Netherlands/5/2008	H13N8	MF145989
A/Black-headed Gull/Netherlands/51/2008	H13N8	MF147023
A/Black-headed Gull/Netherlands/52/2008	H13N8	MF145940
A/Black-headed Gull/Netherlands/55/2008	H13N8	KX979227
A/Black-headed Gull/Netherlands/6/2008	H13N8	MF146566
A/Black-headed Gull/Netherlands/64/2008	H13N8	MF147491
A/Black-headed Gull/Netherlands/65/2008	H13N8	MF682781
A/Black-headed Gull/Netherlands/66/2008	H13N8	MF146391
A/Black-headed Gull/Netherlands/67/2008	H13N8	MF146670
A/Black-headed Gull/Netherlands/68/2008	H13N8	MF145978
A/Black-headed Gull/Netherlands/7/2008	H13N8	MF147648
A/Black-headed Gull/Netherlands/70/2008	H13N8	MF146424
A/Black-headed Gull/Netherlands/74/2008	H13N8	MF146501
A/Black-headed Gull/Netherlands/8/2008	H13N8	MF575016
A/Black-headed Gull/Netherlands/83/2008	H13N8	KX979576
A/Black-headed Gull/Netherlands/86/2008	H13N8	MF146676
A/Black-headed Gull/Netherlands/87/2008	H13N8	MF145996
A/Black-headed Gull/Netherlands/88/2008	H13N8	MF147363
A/Black-headed Gull/Netherlands/9/2008	H13N8	KX978340
A/Black-headed Gull/Netherlands/93/2008	H13N8	MF145945
A/Black-headed Gull/Netherlands/94/2008	H13N8	KX978300
A/Black-headed Gull/Netherlands/95/2008	H13N8	MF148068
A/Black-headed Gull/Netherlands/96/2008	H13N8	KX979163
A/Black-headed Gull/Netherlands/97/2008	H13N8	MF147665
A/Great black-backed Gull/Newfoundland/296/2008	H13N2	GU724153
A/Herring Gull/Mongolia/454/2008	H13N8	JF775470
**A/Laughing Gull/AI08-0714/NJ/08**	**H13N9**	**KF612956**
**A/Laughing Gull/AI08-1388/NJ/08**	**H13N9**	**KF612957**
**A/Laughing Gull/AI08-1460/NJ/08**	**H13N9**	**KF612958**
A/Black-headed Gull/Netherlands/1/2009	H13N2	KX979507
A/Black-headed Gull/Netherlands/11/2009	H13N6	KX979019
A/Black-headed Gull/Netherlands/13/2009	H13N2	MF145916
A/Black-headed Gull/Netherlands/17/2009	H13N3	KX978076
A/Black-headed Gull/Netherlands/18/2009	H13N2	KX978980
A/Black-headed Gull/Netherlands/2/2009	H13N2	MF147797
**A/Black-headed Gull/Netherlands/20/2009**	**H13N2**	**KR087598**
A/Black-headed Gull/Netherlands/27/2009	H13N2	KX978876
A/Black-headed Gull/Netherlands/29/2009	H13N6	KX979544
A/Black-headed Gull/Netherlands/3/2009	H13N2	MF146414
A/Black-headed Gull/Netherlands/31/2009	H13N2	KX979380
A/Black-headed Gull/Netherlands/33/2009	H13N2	KX978020
A/Black-headed Gull/Netherlands/34/2009	H13N2	MF682844
A/Black-headed Gull/Netherlands/36/2009	H13N2	MF147594
A/Black-headed Gull/Netherlands/37/2009	H13N2	KX978043

Virus	Subtype	Accession number
A/Black-headed Gull/Netherlands/38/2009	H13N2	MF148103
A/Black-headed Gull/Netherlands/39/2009	H13N6	KX979208
A/Black-headed Gull/Netherlands/4/2009	H13N2	MF959989
A/Black-headed Gull/Netherlands/5/2009	H13N2	MF146421
A/Black-headed Gull/Netherlands/6/2009	H13N2	MF147945
**A/Black-headed Gull/Netherlands/7/2009**	**H13N2**	**KR087564**
A/Black-headed Gull/Netherlands/8/2009	H13N2	MF147869
A/Black-headed Gull/Netherlands/9/2009	H13N2	KX978293
A/Black-legged Kittiwake/Quebec/02838-1/2009	H13	CY125301
A/Common Gull/Norway/101313/2009	H13N2	HE802715
A/Glaucous-winged Gull/SouthcentralAlaska/9JR0691R1/2009	H13N6	CY070850
A/Glaucous-winged Gull/SouthcentralAlaska/9JR0738R1/2009	H13N6	CY070858
A/Glaucous-winged Gull/SouthcentralAlaska/9JR0747R1/2009	H13N6	CY070866
A/Glaucous-winged Gull/SouthcentralAlaska/9JR0769R1/2009	H13N6	CY070874
A/Glaucous-winged Gull/SouthcentralAlaska/9JR0781R1/2009	H13N6	CY070882
A/Glaucous-winged Gull/SoutheasternAlaska/9JR0822R0/2009	H13N6	CY130340
A/Gull/Delaware/AI09-435/2009	H13-mixed	CY145987
A/Hooded Merganser/NewBrunswick/3750/2009	H13	CY125309
A/Mallard Black Duck hybrid/NewBrunswick/3736/2009	H13N6	CY128958
A/Ring-billed Gull/Quebec/02622-1/2009	H13-mixed	CY125317
**A/Ruddy Turnstone/AI09-294/NJ/09**	**H13N6**	**KF612950**
**A/Shorebird/DE/204/2009**	**H13N6**	**KF612949**
A/Slender-billed gull/CHBZ/11/2009	H13N2	KU684463
A/Black-headed Gull/Netherlands/1/2010	H13N6	KX977814
A/Black-headed Gull/Netherlands/10/2010	H13N8	KX978155
A/Black-headed Gull/Netherlands/12/2010	H13N2	KX978913
A/Black-headed Gull/Netherlands/13/2010	H13N2	KX979541
A/Black-headed Gull/Netherlands/2/2010	H13N8	MF146087
A/Black-headed Gull/Netherlands/4/2010	H13N8	KX977869
A/Black-headed Gull/Netherlands/5/2010	H13N8	MF145955
A/Black-headed Gull/Netherlands/6/2010	H13N8	KX979279
A/Black-headed Gull/Netherlands/7/2010	H13N8	MF682666
A/Black-headed Gull/Netherlands/8/2010	H13N2	KX979026
A/Mallard/Korea/SH38-45/2010	H13	JX030406
**A/Ring-billed Gull/AI10-1708/MN/10**	**H13N6**	**KF612953**
A/Yellow-legged Gull/Georgia/1/2010	H13N2	KC541676
A/Black-headed Gull/Georgia/1/2011	H13N8	KC541677
A/Black-headed Gull/Georgia/3/2011	H13N8	KC541680
A/Black-headed Gull/Georgia/6/2011	H13N8	KC541682
A/Black-headed Gull/Georgia/7/2011	H13N6	KC541687
A/Black-headed Gull/Netherlands/10/2011	H13N8	MF147406
A/Black-headed Gull/Netherlands/12/2011	H13N8	MF146180
A/Black-headed Gull/Netherlands/14/2011	H13N8	MF147288
A/Black-headed Gull/Netherlands/15/2011	H13N8	KX979066
**A/Black-headed Gull/Netherlands/16/2011**	**H13N8**	**KR087588**
**A/Black-headed Gull/Netherlands/17/2011**	**H13N8**	**KR087589**
A/Black-headed Gull/Netherlands/18/2011	H13N8	KX977693
A/Black-headed Gull/Netherlands/19/2011	H13N8	MF145917
A/Black-headed Gull/Netherlands/2/2011	H13N8	KX979383
**A/Black-headed Gull/Netherlands/20/2011**	**H13N8**	**KR087590**
A/Black-headed Gull/Netherlands/21/2011	H13N8	KX978666
A/Black-headed Gull/Netherlands/23/2011	H13N8	MF575221
A/Black-headed Gull/Netherlands/24/2011	H13N8	MF575295
A/Black-headed Gull/Netherlands/25/2011	H13N8	MF147953
**A/Black-headed Gull/Netherlands/26/2011**	**H13N8**	**KR087591**
**A/Black-headed Gull/Netherlands/3/2011**	**H13N8**	**KR087583**
**A/Black-headed Gull/Netherlands/35/2011**	**H13N8**	**KR087592**
**A/Black-headed Gull/Netherlands/36/2011**	**H13N8**	**KR087593**
**A/Black-headed Gull/Netherlands/38/2011**	**H13N8**	**KR087594**

Virus	Subtype	Accession number
**A/Black-headed Gull/Netherlands/39/2011**	**H13N8**	**KR087595**
**A/Black-headed Gull/Netherlands/4/2011**	**H13N8**	**KR087584**
**A/Black-headed Gull/Netherlands/5/2011**	**H13N8**	**KR087585**
**A/Black-headed Gull/Netherlands/8/2011**	**H13N3**	**KR087586**
**A/Black-headed Gull/Netherlands/9/2011**	**H13N8**	**KR087587**
A/Duck/Interior Alaska/11PG00703/2011	H13-mixed	CY195637
A/Glaucous-winged Gull/Southcentral Alaska/11JR02182/2011	H13-mixed	CY195628
A/Glaucous-winged Gull/Southcentral Alaska/11JR02474/2011	H13N6	CY196611
A/Yellow-legged Gull/Georgia/1/2011	H13N6	KC541688
A/Yellow-legged Gull/Georgia/1/2011	H13N8	MF146200
A/Black-headed Gull/Georgia/5/2012	H13N6	MF682848
A/Black-headed Gull/Netherlands/1/2012	H13N6	MF146992
A/Black-headed Gull/Netherlands/10/2012	H13N6	MF147689
A/Black-headed Gull/Netherlands/100/2012	H13N6	KX978985
A/Black-headed Gull/Netherlands/101/2012	H13N6	MF145970
A/Black-headed Gull/Netherlands/102/2012	H13N6	KX977943
A/Black-headed Gull/Netherlands/103/2012	H13N6	MF147266
A/Black-headed Gull/Netherlands/104/2012	H13N6	KX979504
A/Black-headed Gull/Netherlands/105/2012	H13N6	KX979468
A/Black-headed Gull/Netherlands/106/2012	H13N6	KX978718
A/Black-headed Gull/Netherlands/108/2012	H13N6	MF146166
A/Black-headed Gull/Netherlands/109/2012	H13N6	KX978793
A/Black-headed Gull/Netherlands/11/2012	H13N6	MF148049
A/Black-headed Gull/Netherlands/110/2012	H13N6	KX978037
A/Black-headed Gull/Netherlands/111/2012	H13N6	MF145899
A/Black-headed Gull/Netherlands/112/2012	H13N6	KX978433
A/Black-headed Gull/Netherlands/113/2012	H13N6	KX979591
A/Black-headed Gull/Netherlands/115/2012	H13N6	KX978183
A/Black-headed Gull/Netherlands/117/2012	H13N6	KX978539
A/Black-headed Gull/Netherlands/118/2012	H13N6	MF147977
A/Black-headed Gull/Netherlands/119/2012	H13N6	MF147208
A/Black-headed Gull/Netherlands/12/2012	H13N6	MF147771
A/Black-headed Gull/Netherlands/120/2012	H13N6	MF146637
A/Black-headed Gull/Netherlands/121/2012	H13N6	KX978308
A/Black-headed Gull/Netherlands/122/2012	H13N6	MF146479
A/Black-headed Gull/Netherlands/123/2012	H13N6	MF146534
A/Black-headed Gull/Netherlands/124/2012	H13N6	MF147740
A/Black-headed Gull/Netherlands/125/2012	H13N6	MF146435
A/Black-headed Gull/Netherlands/126/2012	H13N6	KX979088
A/Black-headed Gull/Netherlands/127/2012	H13N6	MF147719
A/Black-headed Gull/Netherlands/128/2012	H13N6	MF147553
A/Black-headed Gull/Netherlands/129/2012	H13N6	MF146833
A/Black-headed Gull/Netherlands/13/2012	H13N6	MF146523
A/Black-headed Gull/Netherlands/130/2012	H13N6	MF147007
A/Black-headed Gull/Netherlands/131/2012	H13N6	MF145735
A/Black-headed Gull/Netherlands/132/2012	H13N6	MF147533
A/Black-headed Gull/Netherlands/133/2012	H13N6	MF147558
A/Black-headed Gull/Netherlands/134/2012	H13N6	KX977630
A/Black-headed Gull/Netherlands/135/2012	H13N6	MF146431
A/Black-headed Gull/Netherlands/136/2012	H13N6	MF146500
A/Black-headed Gull/Netherlands/137/2012	H13N6	MF147405
A/Black-headed Gull/Netherlands/138/2012	H13N6	MF146583
A/Black-headed Gull/Netherlands/14/2012	H13N6	MF147101
A/Black-headed Gull/Netherlands/15/2012	H13N6	MF147814
A/Black-headed Gull/Netherlands/16/2012	H13N6	MF145897
A/Black-headed Gull/Netherlands/17/2012	H13N6	MF147189
A/Black-headed Gull/Netherlands/18/2012	H13N6	MF147647
A/Black-headed Gull/Netherlands/19/2012	H13N6	MF148130
A/Black-headed Gull/Netherlands/2/2012	H13N6	KX977772

Virus	Subtype	Accession number
A/Black-headed Gull/Netherlands/20/2012	H13N6	MF146063
A/Black-headed Gull/Netherlands/21/2012	H13N6	MF147878
A/Black-headed Gull/Netherlands/22/2012	H13N6	MF145799
A/Black-headed Gull/Netherlands/23/2012	H13N6	KX977887
A/Black-headed Gull/Netherlands/24/2012	H13N6	MF147679
A/Black-headed Gull/Netherlands/25/2012	H13N6	KX977702
A/Black-headed Gull/Netherlands/26/2012	H13N6	MF147342
A/Black-headed Gull/Netherlands/27/2012	H13N6	MF146953
A/Black-headed Gull/Netherlands/28/2012	H13N6	MF147705
A/Black-headed Gull/Netherlands/29/2012	H13N6	MF146506
A/Black-headed Gull/Netherlands/3/2012	H13N6	MF146473
A/Black-headed Gull/Netherlands/30/2012	H13N6	MF146800
A/Black-headed Gull/Netherlands/31/2012	H13N6	MF147302
A/Black-headed Gull/Netherlands/32/2012	H13N6	MF146858
A/Black-headed Gull/Netherlands/33/2012	H13N6	KX979200
A/Black-headed Gull/Netherlands/34/2012	H13N6	MF146167
A/Black-headed Gull/Netherlands/35/2012	H13N6	MF147197
A/Black-headed Gull/Netherlands/36/2012	H13N6	MF147122
A/Black-headed Gull/Netherlands/37/2012	H13N6	MF147911
A/Black-headed Gull/Netherlands/38/2012	H13N6	MF147940
A/Black-headed Gull/Netherlands/39/2012	H13N6	MF146672
A/Black-headed Gull/Netherlands/4/2012	H13N6	MF146383
A/Black-headed Gull/Netherlands/40/2012	H13N6	MF147673
A/Black-headed Gull/Netherlands/41/2012	H13N6	MF145921
A/Black-headed Gull/Netherlands/42/2012	H13N6	KX978470
A/Black-headed Gull/Netherlands/43/2012	H13N6	MF147698
A/Black-headed Gull/Netherlands/44/2012	H13N6	MF146594
A/Black-headed Gull/Netherlands/45/2012	H13N6	MF146496
A/Black-headed Gull/Netherlands/46/2012	H13N6	MF145843
A/Black-headed Gull/Netherlands/47/2012	H13N6	KX978071
A/Black-headed Gull/Netherlands/48/2012	H13N6	MF147537
A/Black-headed Gull/Netherlands/49/2012	H13N6	MF146211
A/Black-headed Gull/Netherlands/5/2012	H13N6	MF146395
A/Black-headed Gull/Netherlands/50/2012	H13N6	MF146634
A/Black-headed Gull/Netherlands/51/2012	H13N6	MF148145
A/Black-headed Gull/Netherlands/52/2012	H13N6	KX977670
A/Black-headed Gull/Netherlands/53/2012	H13N6	MF147610
A/Black-headed Gull/Netherlands/54/2012	H13N6	MF145811
A/Black-headed Gull/Netherlands/55/2012	H13N6	MF147656
A/Black-headed Gull/Netherlands/56/2012	H13N6	MF147422
A/Black-headed Gull/Netherlands/57/2012	H13N6	MF147944
A/Black-headed Gull/Netherlands/58/2012	H13N6	MF146043
A/Black-headed Gull/Netherlands/59/2012	H13N6	MF147717
A/Black-headed Gull/Netherlands/6/2012	H13N6	MF146975
A/Black-headed Gull/Netherlands/60/2012	H13N6	MF147750
A/Black-headed Gull/Netherlands/61/2012	H13N6	MF147841
A/Black-headed Gull/Netherlands/62/2012	H13N6	MF146710
A/Black-headed Gull/Netherlands/63/2012	H13N6	MF146214
A/Black-headed Gull/Netherlands/64/2012	H13N6	MF147469
A/Black-headed Gull/Netherlands/65/2012	H13N6	KX977811
A/Black-headed Gull/Netherlands/66/2012	H13N6	MF146872
A/Black-headed Gull/Netherlands/67/2012	H13N6	MF145901
A/Black-headed Gull/Netherlands/68/2012	H13N6	MF146441
A/Black-headed Gull/Netherlands/69/2012	H13N6	KX978831
A/Black-headed Gull/Netherlands/7/2012	H13N6	MF145876
A/Black-headed Gull/Netherlands/70/2012	H13N6	MF146204
A/Black-headed Gull/Netherlands/71/2012	H13N6	MF145709
A/Black-headed Gull/Netherlands/72/2012	H13N6	KX978101
A/Black-headed Gull/Netherlands/73/2012	H13N6	MF147965

Virus	Subtype	Accession number
A/Black-headed Gull/Netherlands/74/2012	H13N6	MF148047
A/Black-headed Gull/Netherlands/75/2012	H13N6	MF147095
A/Black-headed Gull/Netherlands/76/2012	H13N6	KX978834
A/Black-headed Gull/Netherlands/77/2012	H13N6	MF146562
A/Black-headed Gull/Netherlands/78/2012	H13N6	MF146693
A/Black-headed Gull/Netherlands/79/2012	H13N6	MF147375
A/Black-headed Gull/Netherlands/8/2012	H13N6	MF146964
A/Black-headed Gull/Netherlands/80/2012	H13N6	MF147078
A/Black-headed Gull/Netherlands/81/2012	H13N6	KX977728
A/Black-headed Gull/Netherlands/82/2012	H13N6	KX979079
A/Black-headed Gull/Netherlands/83/2012	H13N6	KX977618
A/Black-headed Gull/Netherlands/84/2012	H13N6	MF146502
A/Black-headed Gull/Netherlands/85/2012	H13N6	KX979040
A/Black-headed Gull/Netherlands/86/2012	H13N6	MF147754
A/Black-headed Gull/Netherlands/87/2012	H13N6	KX979045
A/Black-headed Gull/Netherlands/88/2012	H13N6	KX978218
A/Black-headed Gull/Netherlands/89/2012	H13N6	MF146309
A/Black-headed Gull/Netherlands/9/2012	H13N6	MF575308
A/Black-headed Gull/Netherlands/90/2012	H13N6	MF147768
A/Black-headed Gull/Netherlands/91/2012	H13N6	MF147484
A/Black-headed Gull/Netherlands/92/2012	H13N6	KX978027
A/Black-headed Gull/Netherlands/94/2012	H13N6	MF682816
A/Black-headed Gull/Netherlands/95/2012	H13N6	MF146344
A/Black-headed Gull/Netherlands/96/2012	H13N6	MF147573
A/Black-headed Gull/Netherlands/97/2012	H13N6	MF147559
A/Black-headed Gull/Netherlands/98/2012	H13N6	KX978937
A/Black-headed Gull/Netherlands/99/2012	H13N6	KX978911
A/Black-headed Gull/Republic of Georgia/2/2012	H13N6	CY185569
A/Duck/Hokkaido/W345/2012	H13N2	LC336769
A/Duck/Hokkaido/WZ68/2012	H13N2	AB812744
A/Gull/Massachusetts/12JR00671/2012	H13N6	CY195663
A/Mediterranean gull/Netherlands/1/2012	H13N6	MF147925
A/Ring-billed Gull/Massachusetts/12DC00060/2012	H13N6	CY195647
A/Ruddy Turnstone/New Jersey/AI12-1737/2012	H13N6	MH501657
A/Yellow-legged Gull/Georgia/4/2012	H13N8	MF147792
A/Yellow-legged Gull/Republic of Georgia/1/2012	mixed	CY185371
A/Yellow-legged Gull/Republic of Georgia/2/2012	H13N6	CY185601
A/Yellow-legged Gull/Republic of Georgia/3/2012	H13N6	CY185609
A/Yellow-legged Gull/Republic of Georgia/5/2012	H13N6	CY185673
A/Yellow-legged Gull/Republic of Georgia/6/2012	H13N6	CY185665
A/Black-headed Gull/Netherlands/10/2013	H13N8	KX977721
A/Black-headed Gull/Netherlands/6/2013	H13N8	MF146245
A/Black-headed Gull/Netherlands/7/2013	H13N8	KX977868
A/Black-headed Gull/Netherlands/8/2013	H13N8	MF147858
A/Black-headed Gull/Netherlands/9/2013	H13N8	MF146285
A/Glaucous-winged Gull/Alaska/387/2013	H13N2	KY131017
A/Glaucous-winged Gull/Alaska/410/2013	H13-mixed	KY131033
A/Glaucous-winged Gull/Alaska/414/2013	H13N2	KY131040
A/Glaucous-winged Gull/Alaska/458/2013	H13N2	KY131056
A/Glaucous-winged Gull/Alaska/544/2013	H13N2	KY131072
A/Glaucous-winged Gull/Alaska/545/2013	H13N2	KY131080
A/Glaucous-winged Gull/Alaska/654/2013	H13N2	KY131112
A/Glaucous-winged Gull/Alaska/660/2013	H13N2	KY131120
A/Glaucous-winged Gull/Alaska/664/2013	H13N2	KY131128
A/Glaucous-winged Gull/Alaska/667/2013	H13N2	KY131136
A/Glaucous-winged Gull/Alaska/695/2013	H13N2	KY131144
A/Gull/Massachusetts/13JR03320/2013	H13N6	CY195825
A/Ring-billed Gull/Massachusetts/13DC30736-1/2013	H13N8	CY195801
A/Ring-billed Gull/Massachusetts/13DC30736-2/2013	H13N8	CY195809

Virus	Subtype	Accession number
A/Yellow-legged Gull/Georgia/1/2013	H13N8	CY185625
A/Yellow-legged Gull/Georgia/2/2013	H13N8	CY185633
A/Black-headed Gull/Netherlands/1/2014	H13N2	KX978817
A/Black-headed Gull/Netherlands/10/2014	H13N6	KX979051
A/Black-headed Gull/Netherlands/11/2014	H13N6	KX977664
A/Black-headed Gull/Netherlands/12/2014	H13N2	KX978367
A/Black-headed Gull/Netherlands/13/2014	H13N2	MF147040
A/Black-headed Gull/Netherlands/14/2014	H13N6	MF147981
A/Black-headed Gull/Netherlands/15/2014	H13N6	KX978686
A/Black-headed Gull/Netherlands/16/2014	H13N6	MF146531
A/Black-headed Gull/Netherlands/17/2014	H13N6	KX979090
A/Black-headed Gull/Netherlands/2/2014	H13N2	MF145726
A/Black-headed Gull/Netherlands/23/2014	H13N2	KX978812
A/Black-headed Gull/Netherlands/24/2014	H13N2	MF147919
A/Black-headed Gull/Netherlands/25/2014	H13N2	KX978026
A/Black-headed Gull/Netherlands/26/2014	H13N6	KX978072
A/Black-headed Gull/Netherlands/27/2014	H13N2	KX977853
A/Black-headed Gull/Netherlands/28/2014	H13N6	KX979165
A/Black-headed Gull/Netherlands/29/2014	H13N6	KX978504
A/Black-headed Gull/Netherlands/3/2014	H13N6	KX977620
A/Black-headed Gull/Netherlands/30/2014	H13N6	MF146090
A/Black-headed Gull/Netherlands/31/2014	H13N6	MF575089
A/Black-headed Gull/Netherlands/32/2014	H13N6	KX978441
A/Black-headed Gull/Netherlands/33/2014	H13N6	MF575196
A/Black-headed Gull/Netherlands/34/2014	H13N6	MF575309
A/Black-headed Gull/Netherlands/35/2014	H13N6	KX978735
A/Black-headed Gull/Netherlands/37/2014	H13N2	MF148148
A/Black-headed Gull/Netherlands/4/2014	H13N2	MF575052
A/Black-headed Gull/Netherlands/5/2014	H13N6	KX978369
A/Black-headed Gull/Netherlands/6/2014	H13N2	KX978977
A/Black-headed Gull/Netherlands/7/2014	H13N6	MF147493
A/Black-headed Gull/Netherlands/8/2014	H13N2	KX978275
A/Black-headed Gull/Netherlands/9/2014	H13N2	MF146305
A/Eurasian curlew/Liaoning/ZH-186/2014	H13N6	KR010435
A/Eurasian curlew/Liaoning/ZH-385/2014	H13N8	KR010443
A/Ruddy Turnstone/New Jersey/UGAI14-1436/2014	H13N6	MH502664
A/Glaucous-winged Gull/Alaska/UGAI15-6732/2015	H13-mixed	KY131286
A/Glaucous-winged Gull/Alaska/UGAI15-6732/2015	H13-mixed	KY131287
A/Glaucous-winged Gull/Southcentral Alaska/15MB01429/2015	H13N6	CY213628
A/Glaucous-winged Gull/Southcentral Alaska/15MB01557/2015	H13N6	CY213620
A/Glaucous-winged Gull/Southcentral Alaska/15MB01610/2015	H13N6	CY213527
A/Glaucous-winged Gull/Southcentral Alaska/15MB01632/2015	H13N6	CY213636
A/Glaucous-winged Gull/Southcentral Alaska/15MB01645/2015	H13N6	CY213535
A/Glaucous-winged Gull/Southcentral Alaska/15MB01667/2015	H13-mixed	CY213644
A/Glaucous-winged Gull/Southcentral Alaska/15MB01693/2015	H13N6	CY213543
A/Glaucous-winged Gull/Southcentral Alaska/15MB01694/2015	H13N6	CY213663
A/Glaucous-winged Gull/Southcentral Alaska/15MB01776/2015	H13N6	CY213687
A/Glaucous-winged Gull/Southcentral Alaska/15MB02016/2015	H13N8	CY213695
A/Glaucous-winged Gull/Southcentral Alaska/15MB02018/2015	H13N8	CY213703
A/Gull/New Jersey/UGAI15-3767/2015	H13N3	MH501054
A/American oystercatcher/Chile/C20102/2016	H13N9	MH499091
A/Black skimmer/Chile/C20057/2016	H13N8	MH498778
A/Black skimmer/Chile/C20077/2016	H13N9	MH499127
A/Black skimmer/Chile/C20084/2016	H13N9	MH498752
A/Black skimmer/Chile/C20100/2016	H13N9	MH499241
A/Black skimmer/Chile/C20108/2016	H13N9	MH499144
A/Black skimmer/Chile/C20123/2016	H13N9	MH499019
A/Black skimmer/Chile/C20124/2016	H13N9	MH498871
A/Black skimmer/Chile/C20140/2016	H13N9	MH499102

Virus	Subtype	Accession number
A/Black skimmer/Chile/C20142/2016	H13N9	MH499225
A/Black-headed Gull/Netherlands/10/2016	H13N2	MF694241
A/Black-headed Gull/Netherlands/11/2016	H13N2	MF694057
A/Black-headed Gull/Netherlands/2/2016	H13N2	MF694026
A/Black-headed Gull/Netherlands/6/2016	H13N2	MF694199
A/Black-headed Gull/Netherlands/7/2016	H13N2	MF694207
A/Black-headed Gull/Netherlands/8/2016	H13N2	MF693954
A/Black-headed Gull/Netherlands/9/2016	H13N2	MF694046
A/Black-tailed Gull/Weihai/115/2016	H13N2	MF461180
A/Black-tailed Gull/Weihai/17/2016	H13N8	MF461188
A/Black-tailed Gull/Weihai/42/2016	H13N2	MH201562
A/Blackish oystercatcher/Chile/C20062/2016	H13N9	MH498730
A/Brown-hooded gull/Chile/C10246/2016	H13N2	MH499037
A/Elegant tern/Chile/C20085/2016	H13N9	MH499011
A/Elegant tern/Chile/C20093/2016	H13N9	MH499177
A/Franklin's gull/Chile/C17421/2016	H13N9	MH498978
A/Franklin's gull/Chile/C17422/2016	H13N9	MH499057
A/Franklin's gull/Chile/C20061/2016	H13N9	MH498647
A/Franklin's gull/Chile/C20069/2016	H13N9	MH498793
A/Franklin's gull/Chile/C20070/2016	H13N9	MH498930
A/Franklin's gull/Chile/C20086/2016	H13N9	MH499107
A/Franklin's gull/Chile/C20094/2016	H13N9	MH499041
A/Franklin's gull/Chile/C20110/2016	H13N9	MH499155
A/Franklin's gull/Chile/C20118/2016	H13N9	MH499132
A/Franklin's gull/Chile/C20149/2016	H13N9	MH499078
A/Franklin's gull/Chile/C20373/2016	H13N9	MH499160
A/Gull/Arica/71/2016	H13N2	MF099262
A/Kelp gull/Chile/C20137/2016	H13N9	MH498851
A/Kelp gull/Chile/C8594/2016	H13N2	MH499186
A/Kelp gull/Chile/C8595/2016	H13N2	MH498919
A/Kelp gull/Chile/C8599/2016	H13N2	MH499244
A/Kelp gull/Chile/C8602/2016	H13N2	MH498862
A/Kelp gull/Chile/C8609/2016	H13N2	MH498888
A/Kelp gull/Chile/C8939/2016	H13N2	MH498698
A/Sandpiper/Southcentral Alaska/16MB01145/2016	H13-mixed	CY213503
A/Shorebird/Chile/C7037/2016	H13N2	MH499147
A/Whimbrel/Chile/C20073/2016	H13N9	MH499218
A/Whimbrel/Chile/C20075/2016	H13N9	MH498668
A/Whimbrel/Chile/C20106/2016	H13N9	MH498656
A/Whimbrel/Chile/C20144/2016	H13N9	MH499009
A/Whimbrel/Chile/C20147/2016	H13N9	MH499072
A/White-backed stilt/Chile/C20090/2016	H13N9	MH498671
A/Kelp gull/Chile/C27733/2017	H13N8	MH499142
A/Laughing Gull/New Jersey/UGAI17-2839/2017	H13N6	MH068335
A/Laughing Gull/New Jersey/UGAI17-2843/2017	H13N6	MH068343
A/Laughing Gull/New Jersey/UGAI17-2850/2017	H13N6	MH068359
A/Laughing Gull/New Jersey/UGAI17-2856/2017	H13N6	MH068367

Table S3

Virus	Subtype	Accession number
A/Black-legged Kittiwake/Alaska/295/1975	H16N3	CY015160
A/Black-headed Gull/Turkmenistan/13/1976	H16N3	EU293864
A/Slender-billed Gull/Astrakhan/28/1976	H16N3	EU293865
**A/Gull/MD/4985/79**	**H16-mixed**	**KF612964**
A/LittleTern/Gurjev/779/1983	H16N3	EU148601
A/Mallard/Gurjev/785/1983	H16N3	EU148600
A/Fulica atra/Volga/635/1986	H16N3	EU564109
A/Herring Gull/NewJersey/780/1986	H16N3	CY136590
A/Shorebird/NewJersey/840/1986	H16N3	CY014599
A/Teal/Volga/671/1986	H16N3	EU148602
A/Herring Gull/DelawareBay/2617/1987	H16N3	CY136606
A/Laughing Gull/DelawareBay/2623/1987	H16N3	CY136614
A/Laughing Gull/DelawareBay/2839/1987	H16N3	CY136630
A/Herring Gull/Delaware/712/1988	H16N3	CY136729
**A/Herring Gull/NJ/163/90**	**H16-mixed**	**KF612962**
**A/Gull/NJ/48/92**	**H16-mixed**	**KF612961**
A/Laughing Gull/DelawareBay/296/1998	H16N3	CY127445
A/Black-headed Gull/Sweden/2/1999	H16N3	AY684888
A/Black-headed Gull/Sweden/3/1999	H16N3	AY684889
A/Black-headed Gull/Sweden/4/1999	H16N3	AY684890
A/Black-headed Gull/Sweden/5/1999	H16N3	AY684891
A/Herring Gull/NewYork/AI00-532/2000	H16N3	CY144178
**A/Waterfowl/GA/96623-7/01**	**H16N3**	**KF612963**
A/Gull/Denmark/68110/2002	H16N3	GQ247872
**A/Black-headed Gull/Sweden/9476/2005**	**H16N3**	**KR087605**
**A/Black-headed Gull/Sweden/9478/2005**	**H16N3**	**MK027211**
**A/Black-headed Gull/Sweden/9479/2005**	**H16N3**	**MK027212**
**A/Black-headed Gull/Sweden/9492/2005**	**H16N3**	**KR087606**
**A/Black-headed Gull/Sweden/9502/2005**	**H16N3**	**KR087607**
**A/Black-headed Gull/Sweden/9504/2005**	**H16N3**	**KR087608**
A/Herring Gull/Finland/13022/2005	H16	KX455114
**A/LittleTern/Sweden/8897/2005**	**H16N3**	**KR087604**
A/wildbird/Sweden/1/2005	H16N3	KR087602
A/wildbird/Sweden/2/2005	H16N3	KR087603
A/Black-headed Gull/Mongolia/1756/2006	H16N3	GQ907294
A/Common Gull/Norway/101617/2006	H16N3	FM179755
A/Environment/Alaska/NWRC184854-12/2006	H16N3	CY122507
A/Environment/NewHampshire/NWRC182016-06/2006	H16N3	CY122448
A/Environment/RhodeIsland/NWRC182872-06/2006	H16N3	CY122476
A/Environment/Utah/NWRC184989-18/2006	H16N3	CY122509
A/European Herring Gull/Netherlands/5/2006	H16N3	MF147450
A/Glaucous Gull/Alaska/44198-027/2006	H16N3	HM059998
A/Herring Gull/Norway/101623/2006	H16N3	FM179756
A/Little Tern/Sweden/55316/2006	H16N3	KR087616
A/Shorebird/Delaware/168/2006	H16N3	EU030976
A/Shorebird/Delaware/172/2006	H16N3	CY130110
A/Shorebird/Delaware/195/2006	H16N3	CY045383
**A/Black-headed Gull/Netherlands/1/2007**	**H16N3**	**KR087609**
**A/Black-headed Gull/Netherlands/3/2007**	**H16N3**	**KR087610**
**A/Black-headed Gull/Netherlands/5/2007**	**H16N3**	**KR087611**
A/Black-headed Gull/Netherlands/7/2007	H16N3	KX978760
**A/Black-headed Gull/Netherlands/8/2007**	**H16N3**	**KR087612**
**A/Black-headed Gull/Netherlands/9/2007**	**H16N3**	**KR087613**
**A/Environment/CO/492008/07**	**H16N3**	**KF612965**
A/Black-headed Gull/Netherlands/100/2008	H16N3	MF147121
A/Black-headed Gull/Netherlands/101/2008	H16N3	KX978252
A/Black-headed Gull/Netherlands/26/2008	H16N3	MF147314
A/Black-headed Gull/Netherlands/27/2008	H16N3	MF146654
A/Black-headed Gull/Netherlands/28/2008	H16N3	MF146238

Virus	Subtype	Accession number
A/Black-headed Gull/Netherlands/29/2008	H16N3	MF146193
A/Black-headed Gull/Netherlands/33/2008	H16N3	MF148040
A/Black-headed Gull/Netherlands/36/2008	H16N3	MF147982
A/Black-headed Gull/Netherlands/40/2008	H16N3	MF146828
A/Black-headed Gull/Netherlands/41/2008	H16N3	MF146643
A/Black-headed Gull/Netherlands/42/2008	H16N3	MF145764
A/Black-headed Gull/Netherlands/43/2008	H16N8	MF147250
A/Black-headed Gull/Netherlands/45/2008	H16N3	MF146076
A/Black-headed Gull/Netherlands/46/2008	H16N3	MF146816
A/Black-headed Gull/Netherlands/47/2008	H16N3	MF146537
A/Black-headed Gull/Netherlands/50/2008	H16N3	MF145888
A/Black-headed Gull/Netherlands/53/2008	H16N3	MF146769
A/Black-headed Gull/Netherlands/54/2008	H16N3	MF147728
A/Black-headed Gull/Netherlands/56/2008	H16N3	MF146014
A/Black-headed Gull/Netherlands/57/2008	H16N3	MF146085
A/Black-headed Gull/Netherlands/59/2008	H16N3	MF147239
A/Black-headed Gull/Netherlands/60/2008	H16N3	MF146436
A/Black-headed Gull/Netherlands/61/2008	H16N3	MF682709
A/Black-headed Gull/Netherlands/62/2008	H16N3	MF147547
A/Black-headed Gull/Netherlands/63/2008	H16N3	KX979453
A/Black-headed Gull/Netherlands/69/2008	H16N3	MF145725
A/Black-headed Gull/Netherlands/72/2008	H16N3	MF146541
A/Black-headed Gull/Netherlands/75/2008	H16N3	MF147192
A/Black-headed Gull/Netherlands/76/2008	H16N3	MF146737
A/Black-headed Gull/Netherlands/77/2008	H16N3	MF146931
A/Black-headed Gull/Netherlands/78/2008	H16N3	MF146836
A/Black-headed Gull/Netherlands/79/2008	H16N3	MF147849
A/Black-headed Gull/Netherlands/80/2008	H16N3	MF146060
A/Black-headed Gull/Netherlands/81/2008	H16N3	KX977730
A/Black-headed Gull/Netherlands/82/2008	H16N3	MF146767
A/Black-headed Gull/Netherlands/84/2008	H16N3	MF147070
A/Black-headed Gull/Netherlands/85/2008	H16N3	MF146577
A/Black-headed Gull/Netherlands/89/2008	H16N3	MF148111
A/Black-headed Gull/Netherlands/91/2008	H16N3	MF147657
A/Black-headed Gull/Netherlands/92/2008	H16N3	KX978323
A/Black-headed Gull/Netherlands/98/2008	H16N3	KX979456
A/Black-headed Gull/Netherlands/99/2008	H16N3	KX978955
A/Glaucous-winged Gull/Southcentral Alaska/12NH01265/2008	H16N3	CY196748
A/Glaucous-winged Gull/Southcentral Alaska/12NH01285/2008	H16N3	CY196756
A/Glaucous-winged Gull/Southcentral Alaska/12NH01518/2008	H16N3	CY196764
A/Glaucous-winged Gull/Southcentral Alaska/12NH01538/2008	H16N3	CY196772
A/Glaucous-winged Gull/Southcentral Alaska/12NH01540/2008	H16N3	CY196780
A/Glaucous-winged Gull/Southcentral Alaska/12NH01593/2008	H16N3	CY196788
A/Glaucous-winged Gull/Southcentral Alaska/12NH01600/2008	H16N3	CY196796
A/Glaucous-winged Gull/Southcentral Alaska/12NH01647/2008	H16N3	CY196804
A/Glaucous-winged Gull/Southcentral Alaska/12NH01679/2008	H16N3	CY196812
A/Great black-backed Gull/Netherlands/1/2008	H16N3	MF146805
**A/Black-headed Gull/Netherlands/10/2009**	**H16N3**	**KR087614**
A/Black-headed Gull/Netherlands/12/2009	H16N3	MF147927
A/Black-headed Gull/Netherlands/14/2009	H16N3	KX978997
A/Black-headed Gull/Netherlands/15/2009	H16N3	MF146410
A/Black-headed Gull/Netherlands/16/2009	H16N3	KX978083
A/Black-headed Gull/Netherlands/19/2009	H16N3	MF146148
**A/Black-headed Gull/Netherlands/21/2009**	**H16N3**	**KR087615**
A/Black-headed Gull/Netherlands/22/2009	H16N3	KX978709
A/Black-headed Gull/Netherlands/23/2009	H16N3	MF146268
A/Black-headed Gull/Netherlands/24/2009	H16N3	MF103719
A/Black-headed Gull/Netherlands/25/2009	H16N3	MF147715
**A/Black-headed Gull/Netherlands/26/2009**	**H16N3**	**KR087572**

Virus	Subtype	Accession number
A/Black-headed Gull/Netherlands/28/2009	H16N3	KX977785
A/Black-headed Gull/Netherlands/32/2009	H16N3	KX979111
A/Black-headed Gull/Netherlands/35/2009	H16N3	KX979094
A/Mallard/Quebec/02916-1/2009	H16	CY125606
A/Black-headed Gull/Iceland/713/2010	H16N3	CY138145
A/Black-headed Gull/Netherlands/11/2010	H16N3	KX977650
A/Black-headed Gull/Netherlands/14/2010	H16N3	KX977766
A/Black-headed Gull/Netherlands/3/2010	H16N3	KX978612
A/Black-headed Gull/Netherlands/9/2010	H16N3	KX978651
A/European Herring Gull/Netherlands/1/2010	H16N3	KX979488
A/Glaucous-winged Gull/Southcentral Alaska/10JR01814/2010	H16N3	CY196009
A/Glaucous-winged Gull/SoutheasternAlaska/10JR01572R0/2010	H16N3	CY130493
A/Glaucous-winged Gull/SoutheasternAlaska/10JR01681R0/2010	H16N3	CY130501
A/Glaucous-winged Gull/SoutheasternAlaska/10JR01700R0/2010	H16N3	CY130509
A/Glaucous-winged Gull/SoutheasternAlaska/10JR01811R0/2010	H16N3	CY130517
A/Gull/SoutheasternAlaska/10JR01527R0/2010	H16N3	CY130485
A/Herring Gull/Newfoundland/GR032/2010	H16N3	KC845043
A/Black-headed Gull/Netherlands/1/2011	H16N3	KX978434
A/Black-headed Gull/Netherlands/11/2011	H16N3	MF147513
A/Black-headed Gull/Netherlands/13/2011	H16N3	KX978398
A/Black-headed Gull/Netherlands/22/2011	H16N3	MF575026
A/Black-headed Gull/Netherlands/27/2011	H16N3	MF146675
A/Black-headed Gull/Netherlands/28/2011	H16N3	MF146920
A/Black-headed Gull/Netherlands/29/2011	H16N3	KX979432
A/Black-headed Gull/Netherlands/30/2011	H16N3	MF145738
A/Black-headed Gull/Netherlands/31/2011	H16N3	MF147961
A/Black-headed Gull/Netherlands/32/2011	H16N3	KX979603
A/Black-headed Gull/Netherlands/33/2011	H16N3	MF146352
A/Black-headed Gull/Netherlands/34/2011	H16N3	MF147682
A/Black-headed Gull/Netherlands/37/2011	H16N3	MF146714
A/Black-headed Gull/Netherlands/6/2011	H16N3	MF146111
A/Black-headed Gull/Netherlands/7/2011	H16N3	MF575109
A/Glaucous-winged Gull/Southcentral Alaska/11JR00366/2011	H16N3	CY196403
A/Glaucous-winged Gull/Southcentral Alaska/11JR01368/2011	H16N3	CY196411
A/Glaucous-winged Gull/Southcentral Alaska/11JR01710/2011	H16N3	CY196419
A/Glaucous-winged Gull/Southcentral Alaska/11JR01711/2011	H16N3	CY196427
A/Glaucous-winged Gull/Southcentral Alaska/11JR01712/2011	H16N3	CY196435
A/Glaucous-winged Gull/Southcentral Alaska/11JR01713/2011	H16N3	CY196443
A/Glaucous-winged Gull/Southcentral Alaska/11JR01716/2011	H16N3	CY196451
A/Glaucous-winged Gull/Southcentral Alaska/11JR01719/2011	H16N3	CY196459
A/Glaucous-winged Gull/Southcentral Alaska/11JR01722/2011	H16-mixed	CY195614
A/Glaucous-winged Gull/Southcentral Alaska/11JR01724/2011	H16N3	CY196467
A/Glaucous-winged Gull/Southcentral Alaska/11JR01725/2011	H16N3	CY196475
A/Glaucous-winged Gull/Southcentral Alaska/11JR01732/2011	H16N3	CY196483
A/Glaucous-winged Gull/Southcentral Alaska/11JR01733/2011	H16N3	CY196491
A/Glaucous-winged Gull/Southcentral Alaska/11JR01734/2011	H16N3	CY196499
A/Glaucous-winged Gull/Southcentral Alaska/11JR01736/2011	H16N3	CY196507
A/Glaucous-winged Gull/Southcentral Alaska/11JR01738/2011	H16N3	CY196515
A/Glaucous-winged Gull/Southcentral Alaska/11JR01739/2011	H16N3	CY196523
A/Glaucous-winged Gull/Southcentral Alaska/11JR01761/2011	H16N3	CY196531
A/Glaucous-winged Gull/Southcentral Alaska/11JR01785/2011	H16N3	CY196539
A/Glaucous-winged Gull/Southcentral Alaska/11JR01852/2011	H16N3	CY196547
A/Glaucous-winged Gull/Southcentral Alaska/11JR01859/2011	H16N3	CY196555
A/Glaucous-winged Gull/Southcentral Alaska/11JR01871/2011	H16N3	CY196563
A/Glaucous-winged Gull/Southcentral Alaska/11JR01902/2011	H16N3	CY196571
A/Glaucous-winged Gull/Southcentral Alaska/11JR01906/2011	H16N3	CY196579
A/Glaucous-winged Gull/Southcentral Alaska/11JR01908/2011	H16N3	CY196587
A/Glaucous-winged Gull/Southcentral Alaska/11JR02017/2011	H16N3	CY196595
A/Glaucous-winged Gull/Southcentral Alaska/11JR02272/2011	H16N3	CY196603

Virus	Subtype	Accession number
A/Black-headed Gull/Netherlands/107/2012	H16N3	KX978749
A/Black-headed Gull/Netherlands/114/2012	H16N3	KX977892
A/Black-headed Gull/Netherlands/93/2012	H16N3	KX979159
A/Black-headed Gull/Republic of Georgia/4/2012	H16N3	CY185585
A/Environment/California/1242V/2012	H16N3	CY176997
A/Glaucous-winged Gull/Southcentral Alaska/12MB01573/2012	H16N3	CY195671
A/Glaucous-winged Gull/Southcentral Alaska/12MB01577/2012	H16N3	CY195679
A/Glaucous-winged Gull/Southcentral Alaska/12MB01618/2012	H16N3	CY195687
A/Glaucous-winged Gull/Southcentral Alaska/12MB01620/2012	H16N3	CY195695
A/Glaucous-winged Gull/Southcentral Alaska/12MB01812/2012	H16N3	CY195703
A/Glaucous-winged Gull/Southcentral Alaska/12MB01823/2012	H16N3	CY195711
A/Glaucous-winged Gull/Southcentral Alaska/12NH01263/2012	H16N3	CY195785
A/Glaucous-winged Gull/Southcentral Alaska/12NH01632/2012	H16N3	CY195793
A/Gull/Massachusetts/12JR00662/2012	H16N3	CY195655
A/Black-headed Gull/Netherlands/2/2013	H16N3	KX977836
A/Black-headed Gull/Netherlands/3/2013	H16N3	KX978186
A/Black-headed Gull/Netherlands/4/2013	H16N3	KX979151
A/Black-headed Gull/Netherlands/5/2013	H16N3	KX978693
A/California Gull/California/1196P/2013	H16N3	CY177441
A/Duck/Hokkaido/WZ82/2013	H16N3	AB937721
A/Duck/Hokkaido/WZ82/2013	H16N3	LC339707
A/Glaucous-winged Gull/Alaska/567/2013	H16N3	KY131088
A/Glaucous-winged Gull/Southcentral Alaska/13MB01431/2013	H16N3	CY239288
A/Glaucous-winged Gull/Southcentral Alaska/13MB02410/2013	H16N3	CY239296
A/Glaucous-winged Gull/Southcentral Alaska/13MB02526/2013	H16N3	CY213711
A/Glaucous-winged Gull/Southcentral Alaska/13MB02527/2013	H16N3	CY213719
A/Glaucous-winged Gull/Southcentral Alaska/13MB02558/2013	H16N3	CY213727
A/Glaucous-winged Gull/Southcentral Alaska/13MB02561/2013	H16N3	CY213735
A/Glaucous-winged Gull/Southcentral Alaska/13MB02569/2013	H16N3	CY213743
A/Glaucous-winged Gull/Southcentral Alaska/13MB02582/2013	H16N3	CY213751
A/Glaucous-winged Gull/Southcentral Alaska/13MB02593/2013	H16N3	CY213759
A/Glaucous-winged Gull/Southcentral Alaska/13MB02599/2013	H16N3	CY213767
A/Gull/Massachusetts/13WP00522/2013	H16N3	CY195833
A/Gull/Massachusetts/13WP00539/2013	H16N3	CY195841
A/Laughing Gull/New Jersey/AI13-1937/2013	H16N3	MH501070
A/Ruddy Turnstone/New Jersey/AI13-2872/2013	H16N3	MH502494
A/Black-headed Gull/Netherlands/18/2014	H16N3	MF146608
A/Black-headed Gull/Netherlands/36/2014	H16N3	MF575190
A/European Herring Gull/Netherlands/2/2014	H16N3	MF147061
A/Glaucous-winged Gull/Alaska/915/2014	H16N3	KT338609
A/Glaucous-winged Gull/Southcentral Alaska/14MB00623/2014	H16N3	CY206862
A/Glaucous-winged Gull/Southcentral Alaska/14MB01306/2014	H16N3	CY206870
A/Glaucous-winged Gull/Southcentral Alaska/14MB01318/2014	H16N3	CY206878
A/Glaucous-winged Gull/Southcentral Alaska/14MB01336/2014	H16N3	CY206677
A/Glaucous-winged Gull/Southcentral Alaska/14MB01383/2014	H16N3	CY206886
A/Glaucous-winged Gull/Southcentral Alaska/14MB01392/2014	H16N3	CY206894
A/Glaucous-winged Gull/Southcentral Alaska/14MB01417/2014	H16N3	CY206902
A/Glaucous-winged Gull/Southcentral Alaska/14MB01418/2014	H16N3	CY206910
A/Glaucous-winged Gull/Southcentral Alaska/14MB01422/2014	H16N3	CY206918
A/Glaucous-winged Gull/Southcentral Alaska/14MB01438/2014	H16N3	CY206926
A/Glaucous-winged Gull/Southcentral Alaska/14MB01444/2014	H16N3	CY239304
A/Glaucous-winged Gull/Southcentral Alaska/14MB01615/2014	H16N3	CY206934
A/Glaucous-winged Gull/Southcentral Alaska/14MB01705/2014	H16N3	CY206942
A/Glaucous-winged Gull/Southcentral Alaska/14MB01770/2014	H16N3	CY206950
A/Glaucous-winged Gull/Southcentral Alaska/14MB01819/2014	H16N3	CY206958
A/Glaucous-winged Gull/Southcentral Alaska/14MB01884/2014	H16N3	CY206966
A/Glaucous-winged Gull/Southcentral Alaska/14MB01886/2014	H16N3	CY206974
A/Glaucous-winged Gull/Southcentral Alaska/14MB01893/2014	H16N3	CY206982
A/Glaucous-winged Gull/Southcentral Alaska/14MB01926/2014	H16N3	CY206990

Virus	Subtype	Accession number
A/Glaucous-winged Gull/Southcentral Alaska/14MB01959/2014	H16N3	CY206998
A/Glaucous-winged Gull/Southcentral Alaska/14MB02018/2014	H16N3	CY207006
A/Glaucous-winged Gull/Southcentral Alaska/14MB02081/2014	H16N3	CY207014
A/Glaucous-winged Gull/Southcentral Alaska/14MB02094/2014	H16N3	CY239312
A/Mallard/Alaska/903/2014	H16N3	KT338601
A/Northern pintail/Alaska/886/2014	H16N3	KT338585
A/Black-headed Gull/Netherlands/1/2015	H16N3	MF147650
A/Black-headed Gull/Netherlands/2/2015	H16N3	KX978663
A/Black-headed Gull/Netherlands/3/2015	H16N3	KX978028
A/Black-headed Gull/Netherlands/4/2015	H16N3	KX978525
A/Black-headed Gull/Netherlands/5/2015	H16N3	KX977739
A/European Herring Gull/Netherlands/3/2015	H16N3	MF693968
A/Glaucous-winged Gull/Southcentral Alaska/15MB01680/2015	H16N3	CY213655
A/Glaucous-winged Gull/Southcentral Alaska/15MB01735/2015	H16N3	CY213551
A/Glaucous-winged Gull/Southcentral Alaska/15MB01747/2015	H16N3	CY213671
A/Glaucous-winged Gull/Southcentral Alaska/15MB01758/2015	H16N3	CY213679
A/Gull/New Jersey/UGAI15-3414/2015	H16N3	MH501022
A/Gull/New Jersey/UGAI15-3459/2015	H16N3	MH501038
A/Lesser black-backed Gull/Netherlands/1/2015	H16N3	MF694110
A/Black-headed Gull/Netherlands/1/2016	H16N3	MF694134
A/Black-headed Gull/Netherlands/3/2016	H16N3	MF694124
A/Brown-hooded gull/Chile/C8851/2016	H16N3	MH498904
A/Environment/New Jersey/UGAI16-0787/2016	H16N3	CY240828
A/Environment/New Jersey/UGAI16-0887/2016	H16-mixed	CY240896
A/Environment/New Jersey/UGAI16-1048/2016	H16N3	CY240948
A/Environment/New Jersey/UGAI16-1713/2016	H16N3	CY241634
A/Franklin's gull/Chile/C10784/2016	H16N3	MH134702
A/Franklin's gull/Chile/C10794/2016	H16N3	MH134685
A/Glaucous-winged Gull/Southcentral Alaska/16MB00031/2016	H16N3	CY239376
A/Glaucous-winged Gull/Southcentral Alaska/16MB00033/2016	H16N3	CY239269
A/Glaucous-winged Gull/Southcentral Alaska/16MB02936/2016	H16N3	CY239320
A/Glaucous-winged Gull/Southcentral Alaska/16MB02941/2016	H16N3	CY239328
A/Glaucous-winged Gull/Southcentral Alaska/16MB02942/2016	H16N3	CY239336
A/Glaucous-winged Gull/Southcentral Alaska/16MB02960/2016	H16N3	CY239344
A/Glaucous-winged Gull/Southcentral Alaska/16MB03027/2016	H16N3	CY239352
A/Glaucous-winged Gull/Southcentral Alaska/16MB03039/2016	H16N3	CY239360
A/Glaucous-winged Gull/Southcentral Alaska/16MB03046/2016	H16N3	CY239368
A/Glaucous-winged Gull/Southcentral Alaska/16MB03089/2016	H16N3	CY239384
A/Glaucous-winged Gull/Southcentral Alaska/16MB03160/2016	H16N3	CY239392
A/Sandpiper/Southcentral Alaska/16MB01145/2016	H16-mixed	CY213504
